# Recent Advances in Applications of Fluorescent Perylenediimide and Perylenemonoimide Dyes in Bioimaging, Photothermal and Photodynamic Therapy

**DOI:** 10.3390/ijms24076308

**Published:** 2023-03-27

**Authors:** Oksana Krupka, Piétrick Hudhomme

**Affiliations:** 1 Univ. Angers, Inserm, CNRS, MINT, SFR ICAT, F-49000 Angers, France; 2 Univ. Angers, CNRS, MOLTECH-Anjou, SFR MATRIX, F-49000 Angers, France

**Keywords:** perylenediimide, perylenemonoimide, dyes, fluorescence, bioimaging, photothermal therapy, photodynamic therapy, cancer therapy, theranostics

## Abstract

The emblematic perylenediimide (PDI) motif which was initially used as a simple dye has undergone incredible development in recent decades. The increasing power of synthetic organic chemistry has allowed it to decorate PDIs to achieve highly functional dyes. As these PDI derivatives combine thermal, chemical and photostability, with an additional high absorption coefficient and near-unity fluorescence quantum yield, they have been widely studied for applications in materials science, particularly in photovoltaics. Although PDIs have always been in the spotlight, their asymmetric counterparts, perylenemonoimide (PMI) analogues, are now experiencing a resurgence of interest with new efforts to create architectures with equally exciting properties. Namely, their exceptional fluorescence properties have recently been used to develop novel systems for applications in bioimaging, biosensing and photodynamic therapy. This review covers the state of the art in the synthesis, photophysical characterizations and recently reported applications demonstrating the versatility of these two sister PDI and PMI compounds. The objective is to show that after well-known applications in materials science, the emerging trends in the use of PDI- and PMI-based derivatives concern very specific biomedicinal applications including drug delivery, diagnostics and theranostics.

## 1. Introduction

**Figure 1:**
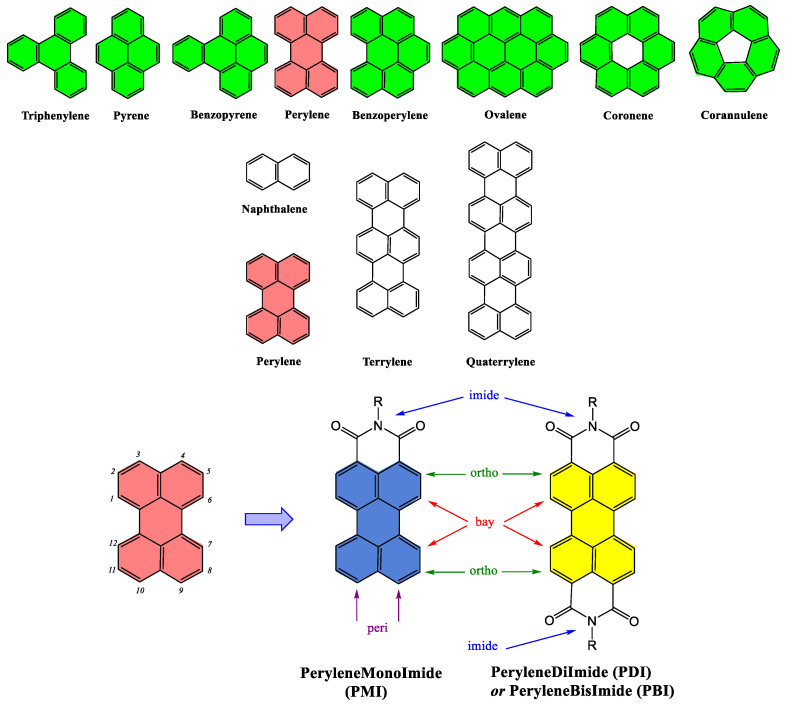
Representation of polycyclic aromatic hydrocarbon (PAH) structures, more specifically the rylene dyes and their emblematic leaders perylenediimide (PDI) or perylenebisimide (PBI) and perylenemonoimide (PMI). In the review, the colour code is as follows: the backbone is coloured green for PAHs, red for perylenes including perylene mono- and dianhydride starting materials, yellow for PDI derivatives and blue for PMI derivatives.

Polycyclic aromatic hydrocarbons (PAHs) are a highly versatile class of organic materials which are constructed through the fusion of benzene units, affording highly extended π-conjugated systems [1,2]. More specifically, in recent years, functionalized PAHs have been designed from triphenylene [3,4], (benzo)pyrene [5,6], (benzo)perylene, ovalene [7], coronene [8,9] or even corannulene [10,11], to mention the main ones that have emerged to serve in organic and materials chemistry (Figure 1). Among them, the perylene core belongs to the family of rylene-based dyes since the rylene framework consists in naphthalene units linked in peri positions, as its counterparts terrylene and quaterrylene are formed by connecting multiple naphthalene units via the peri position [12]. Originally, perylene derivatives were widely used as dyes, but their current application has extended far beyond that, thanks to their unique optical, electrochemical, thermal and luminescent properties. They have recently had a significant impact on the development of organic electronics. More specifically, the rylene imide function spearheaded this development to reach electron acceptors of major interest in energy storage [13] and organic electronics [14], in particular in organic field-effect transistors (OFETs), organic light-emitting diodes (OLEDs) and organic photovoltaic (OPV) devices [15]. Within this family, perylenediimide (PDI) with two electron-withdrawing imide moieties on each side of the perylene moiety and asymmetric perylenemonoimide (PMI) with only one imide group have attracted a great deal of attention from materials chemists in recent decades.

Perylene-3,4:9,10-tetracarboxylic acid diimide derivatives, most commonly known as perylenediimides (PDI) or perylenebisimides (PBI), firstly introduced by Kardos in 1913 [16], were historically applied as textile vat dyes and industrial pigments in paints, lacquers and reprographic processes [17,18]. Over the last three decades, due to the pioneering work of Langhals [19], they have become a staple and one of the most studied dyes, leading to an ever-widening range of applications. Because of their outstanding high chemical, thermal and photochemical stability, their strong visible-light-absorbing capabilities with a high extinction coefficient and quantum yields of fluorescence close to unity, but also their excellent photophysical properties, PDI derivatives are among the best n-type semiconductors available to date, providing advantages for using PDI derivatives in organic electronics. Moreover, the inventiveness of organic chemists to functionalize this PDI backbone has enabled the field of applications to be extended even further. This has led to a significant number of reviews in recent years concerning the synthetic chemistry of PDI derivatives [20,21,22] and their use as building blocks in supramolecular chemistry [23,24] or in self-assembled nanostructures [25], and for the elaboration of strategies for generating chiral architectures from this initial planar system [26]. These materials are also investigated for new applications when linked to fullerene C_60_ [27] or for light-energy conversion in photosynthetic systems [28]. Of course, their high potential as electron-poor arylenediimides [29,30] is still widely exploited today for important developments in organic electronics [31], more specifically in OLEDs [32], OFETs [33] and as nonfullerene acceptors (NFAs), small molecules in organic solar cells (OSCs) [34,35,36,37,38,39,40,41,42] or in all-polymer solar cells (All-PSCs) [43]. Finally, besides the evident use of PDI-based molecules in organic electronic devices, the interest in their applications related to biology has increased progressively. Different topics have been reviewed where PDI derivatives are used as versatile fluorescent tools in environmental and biological analysis [44], or for pollutant detection and degradation [45]. For such bioapplications, PDI derivatives must be able to self-assemble in water [46] or be properly decorated with water-soluble groups [47]. Multifunctional water-soluble PDI derivatives presenting high fluorescence and photostability properties accompanied with necessary biocompatibility were reviewed in 2016, thus highlighting the promising future of PDI-based materials in biological applications [48]. Similarly, synthetic strategies that involve convergent and divergent approaches of PDI-cored dendrimers have been widely discussed, showing their promising development in bioimaging and gene-delivery applications [49]. Recent advances in the use of pyrene- and perylene-based dyes, including PDI and PMI derivatives, have been highlighted as excimer-based fluorescence probes for biological applications [50]. Finally, the recent research progress on PDI nanostructures for advanced phototheranostics [51] in the treatment and diagnosis of diseases based on PDIs was recently summarized [52].

As can be seen here, PDI-based architectures have been at the forefront of the development of this family of perylene. Nevertheless, the asymmetric PMI derivatives, which have been relatively less exploited until now, have recently aroused growing interest among organic chemists to build new architectures with increasingly original properties. Indeed, their history is much more recent, as PMIs were first reported by Langhals in 1995 [53]. In addition to the ever-present possibility of functionalizing the imide, ortho and bay positions, the PMI core presents two unique peri positions. Nevertheless, their high thermal and photostability, outstanding absorption, fluorescence emission and electrochemical properties confirm that PMI derivatives are also particularly attractive for applications in materials science, as recently very interestingly reviewed [54].

The aim of this review is to present the different synthetic methodologies studied in recent years to prepare PDI and PMI derivatives for biological and medicinal applications. More specifically, the aim is to highlight the use of their absorption and emission properties to demonstrate the high potential of these two families of rylene dyes for the development of materials that are highly relevant for bioimaging, photothermal and photodynamic therapies. We approach the different strategies devised by organic chemists to make these polycyclic aromatic derivatives highly soluble in water or solvents compatible with biological and medicinal applications. We have seen that these developments mainly affect the imide and bay positions, with the peri position supplemented for the PMI backbone, considering that the ortho position is, to this day, too difficult to access and little exploited. However, it is worth noting the creative engineering implemented to adapt these solubility properties with the specifications required in terms of absorption or emission to achieve the desired applications.

## 2. Presentation and Requirements for Photodynamic Therapy, Photothermal Therapy and Bioimaging Applications

**Figure 2:**
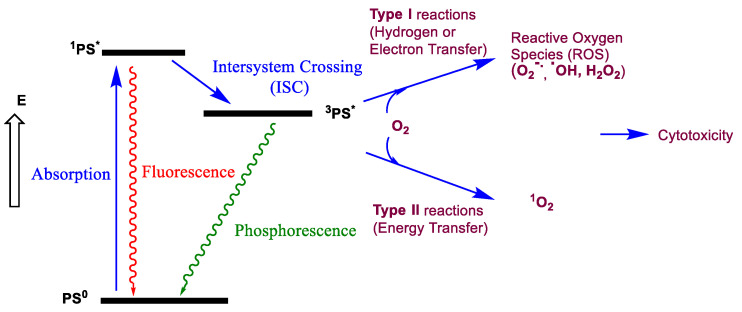
Mechanism of PDT represented by modified Jablonski diagram after administration of the photosensitizer [55,61].

Photodynamic therapy (PDT) has become an increasingly important therapeutic protocol for cancer therapy because it offers selectivity and minimal invasiveness with reduced side effects in comparison to traditional chemotherapy [55]. This therapy, which is already applied for several types of cancers at the clinical level, consists in a two-stage procedure [56]. Firstly, a light-sensitive photosensitizer (PS) is administrated to the patient preferentially in cancer cells over healthy cells. Secondly, a light of appropriate wavelength is applied to this area, causing the PS to access its triplet-excited state ^3^PS* via the intersystem crossing process (ISC) (Figure 2). Following this activation, the PS can transfer its excitation energy via an energy transfer to the surrounding O_2_ molecules to generate reactive singlet oxygen ^1^O_2_ species, a form of oxygen that is highly toxic for cancer cells. In fact, in the presence of oxygen, two competing processes, called type I and type II reactions, can occur on the excited sensitizer [57]. The common ^3^PS* triplet species reacts with O_2_ via electron or hydrogen transfer (type I) to produce reactive oxygen species (ROS) such as superoxide radical (O_2_^−•^), hydroxyl radical (HO^•^) and hydrogen peroxide (H_2_O_2_). The second mechanism (type II) involves an energy transfer process to generate a singlet oxygen (^1^O_2_) species [58,59]. Consequently, apart from light and oxygen, the PS is the crucial element of PDT and must present selectivity towards tumour cells [60].

Today, the PSs approved or in clinical trial are mostly porphyrin precursors with Porfimer sodium (Photofrin), but also aminolevulinic acid, phenothiazines, cyanines (merocyanine), hypericin and xanthenes, which have been considered good candidates [62]. An ideal photosensitizing agent should possess large absorption, low toxicity and high solubility in biocompatible solutions. To achieve the goals for cancer diagnosis and therapy, the structure–property relationships clearly show that the synthetic dyes must exhibit absorption in the near-infrared (NIR) region, particularly in the first biological window (700–1000 nm, NIR-I) and the second biological window (1000–1700 nm, NIR-II) [63] to minimize light scattering and penetrate deeper into the tissue [64,65], Generally, the presence of heavy atoms such as metal atoms (zinc, platinum, palladium), halogen atoms (mostly iodine, bromine) or heteroatoms (O, N, S, P) conjugated to a fluorophore enhances its photodynamic power through triplet-state conversion, corresponding to the ISC, causing direct cytotoxicity by ^1^O_2_ formation. Nevertheless, the strategy to incorporate heavy-metal atoms into photosensitizer structures causes elevated dark toxicity, short triplet-state lifetimes and poor photostability, and depends on the high cost of heavy metals. To address these drawbacks, efforts are devoted to developing advanced smart photosensitizers without the use of heavy atoms to better fit the clinical requirements of PDT [66].

Photothermal therapy (PTT) is a minimally invasive therapeutic treatment that relies on the activation of photosensitizing agents by pulsed NIR laser irradiation to generate heat for the thermal ablation of cancer tumours [67]. An important advantage of this therapy, which involves a nonradiative conversion of light energy into heat, is the deep penetration ability into the tissue accompanied with a minimal effect of nonselective cell death on surrounding healthy tissue. Strategies for developing organic NIR-absorbing molecules for photothermal cancer therapy have drawn intensive attention in recent years. However, the main drawbacks of most of the synthesized molecules concern their photobleaching under light irradiation.

Fluorescence imaging has clearly demonstrated its versatility as a practical method for biological analysis, clinical diagnosis and drug delivery [68]. This technique is implemented in vivo using light excitation in different regions, including the visible region (400–700 nm), the first conventional NIR region (NIR-I, 700–1000 nm) and the second NIR region (NIR-II, 1000–1700 nm) [69,70]. Much effort has been focused on small-molecule fluorescent probes with absorption and emission in the visible region [71]. However, this makes them difficult to use for detection and imaging in live animals, as the absorption and autofluorescence of biomolecules in the UV-Visible region are high. For example, fluorophores requiring blue and/or green excitation light are compatible only for superficial structures because of the poor tissue penetration at such wavelengths. For fluorophores requiring excitation with yellow or red light (around 600 nm), an excessive autofluorescence is observed because endogenous fluorophores, such as haemoglobin, are also excited in this range [72]. As it is known that visible light penetrates little into tissues, there is a general interest in the NIR-I region, defined as the “NIR window of biological transparency”, which allows penetration into these tissues, despite the intrinsic problems of relatively high tissue autofluorescence and unsatisfactory imaging depth. On the contrary, the NIR-II fluorescence imaging improves the depth of penetration into tissues, allowing the visualisation of biological molecules in deep tissues. Therefore, bioimaging requires fluorophores with excellent stability and high fluorescence quantum yield with additional red and NIR emission to avoid biological background fluorescence and allow deep penetration into tissues.

## 3. PDI and PMI Structures: Common Features and Specificities

### 3.1. Structural Elements for the Design of PDI and PMI Derivatives

In order to exploit the particularly interesting properties of PDI and PMI fluorophores, it is first necessary to solve the problem of their low solubility in common solvents. Indeed, this is due to a very strong tendency to aggregate because of the flatness of the perylene skeleton. This drawback becomes even more crucial for biological applications, where the choice in compatible solvents is even more limited. Organic chemists have found a way to overcome these problems of solubility. First, the introduction of branched chains or sterically bulky groups in the imide position block the tendency to aggregate. This is not accompanied by a change in the electronic properties of the PDI and PMI scaffolds due to the presence of a node on the imide nitrogen in the highest occupied (HOMO) and lowest unoccupied (LUMO) molecular orbitals. Secondly, the other possibility to overcome their poor solubility is to introduce bulky and sterically hindered substituents in the ortho or bay positions. In particular, in the bay position, this results in a relatively large twist of the backbone, which strongly limits aggregation. Nevertheless, substitution in the ortho and bay positions significantly alters the electrochemical properties as well as optical and photophysical properties of the ground and excited states. Finally, concerning only the PMI unit, the substitution of the peri position does not contribute significantly to the improvement in solubility, but instead modifies the photophysical and electrochemical properties of the PMI core in solution.

PDI and PMI derivatives share a number of common properties. The unsubstituted perylene skeleton presents an absorption in the visible region (400–550 nm) with high molar-extinction coefficients around 10^4^ M^−1^.cm^−1^. Regarding the emission properties, PMI exhibits a slight bathochromic shift compared to its PDI counterpart, with a comparatively somewhat larger Stokes shift. It is commonly accepted that unsubstituted PDI derivatives in the bay region are not specifically favourable for bioimaging, as the perylene unit fluoresces at about 540 nm with very small Stokes shifts. Therefore, it is necessary to introduce electron-donating substituents such as phenoxy or alkylamino groups in the ortho and bay positions, or in the peri position for PMI, resulting in red shifts of their absorption and emission maxima with a decrease in the accepting character of the PDI and PMI derivatives. In addition, these alkylamino groups often induce more pronounced nonradiative decays, resulting in lower fluorescence quantum yields but higher photodynamic and photothermal efficiencies.

Rylenecarboximide-based molecular structures represented by PMI and PDI—but also terrylenediimide (TDI) and quaterrylenediimide (QDI) [73]—derivatives characterized by an exceptional photostability and absorption and fluorescence properties have been recognized as important tools for biological applications [74]. Initially used for fluorescence imaging, these dyes are now very promising in the field of cancer theranostics, as they combine both diagnostics and therapeutics properties. Indeed, unlike most other dyes considered for such applications, these rylenecarboximide derivatives do not suffer from limited photostability which leads to undesirable photobleaching in a biological environment.

### 3.2. Perylenediimide (PDI)-Based Building Blocks for Designing New Architectures

**Scheme 1:**
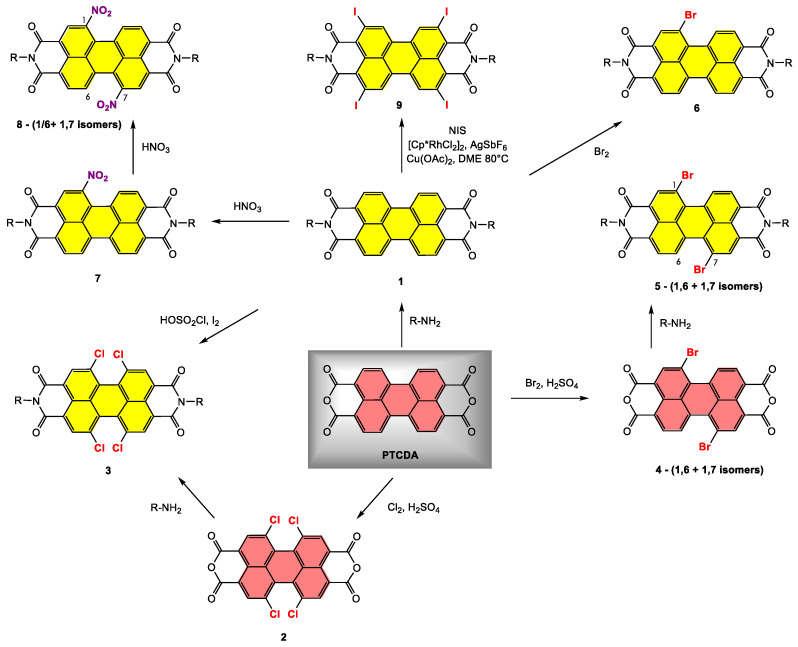
Synthetic methods for preparation of PDI-based key building blocks.

The perylene scaffold presents three different functionalizable positions with the 3,4,9,10, known as the peri positions, corresponding to the diimide functions for PDI derivatives. The introduction of substituents to these imide nitrogen atoms greatly improves the solubility of corresponding materials. The other 1,6,7,12 positions (bay region) and the 2,5,8,11 positions (ortho region) play an essential role in the tuning of their optical and electronic properties. The perylene-3,4:9,10-tetracarboxylic acid dianhydride (PTCDA) compound remains the principal starting material for synthesizing PDI derivatives, and the imidization reaction on PTCDA using an aliphatic or aromatic primary amine results in the formation of symmetrical *N*,*N*-disubstituted PDIs **1** (Scheme 1) [75,76]. While the introduction of solubilizing groups on the imide nitrogen atoms maintains the backbone planarity, the introduction of substituents into the bay region enforces a considerable twisting of the perylene skeleton as a result of electrostatic repulsion and steric effects among the substituents (31.7° for 1,7-dibromo-PDI [77] or 37° for 1,6,7,12-tetrachloro-PDI [78]) by disrupting π–π stacking between PDI units. Moreover, the synthesis of such PDI bay-substituted derivatives has considerably promoted the development of emerging applications thanks to their increased solubility in organic solvents and the possibility to tune their electronic properties [20,79]. Historically, the functionalization of the perylene core has been achieved via the formation of 1,6,7,12-tetrachlorinated or 1-mono- and 1,6/1,7-dibrominated PDIs as the key building blocks of high importance. The tetrachlorination of PTCDA by action of chlorine and sulphuric acid is a straightforward procedure to obtain 1,6,7,12-tetrachloroperylene tetracarboxylic acid dianhydride **2**, which is followed by the imidization step, resulting in compound **3**. Bay-unsubstituted PDI derivative **1** can also be tetrahalogenated into PDI **3** using chlorosulfonic acid and iodine. On the other hand, mono- and dibromo-substituted PDI derivatives are also accessible by a two-step synthesis. First, a two-fold electrophilic substitution of PTCDA is carried out with bromine in concentrated sulphuric acid in the presence of a catalytic amount of iodine, affording a complicated mixture of major 1,7- and minor 1,6-dibromoperylene dianhydride isomers **4** accompanied with some 1,6,7-tribromo compound. Subsequent imidization using primary amines affords the corresponding mixture of isomeric dibromo-PDI derivatives **5** [80]. This bromination reaction can also be carried out on PDI derivative **1** after the imidization step. This reaction suffers from long reaction times and the use of a large excess of bromine. As a result, its poor selectivity results in a mixture of monobromo **6** and bis-bromo PDI **5** derivatives, which requires time-consuming chromatography for purification. Considering that further functionalization of the PDI skeleton requires a nucleophilic aromatic substitution or a metal-catalysed cross-coupling reaction, an interesting leaving group has recently emerged with the introduction of a nitro group in the bay region. More importantly, mononitration is much easier to control and more selective thanks to the electron-withdrawing character of the nitro group. This reaction leading to mononitro-PDI **7** can be carried out in the presence of nitric acid with or without cerium ammonium nitrate (CAN) with a short reaction time, at room temperature, in nearly quantitative yield and in multigram scale [81,82,83,84]. When the reaction is performed with an excess of nitric acid at room temperature, the dinitro-PDI **8** is afforded in high yield as a mixture of 1,6 and 1,7 isomers [81,85]. Finally, concerning halogenation in the ortho position, tetrachlorination and tetrabromination in the 2,5,8,11 positions were described using an intermediate tetraborylated PDI derivative [86,87]. However, a straightforward tetraiodination using N-iodosuccinimide as the iodine source directly yields PDI **9**, which is of great interest for the further development of ortho substitutions [88].

**Scheme 2:**
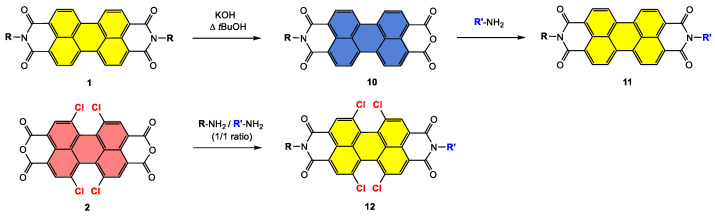
Desymmetrization procedures of the PDI backbone in the imide positions.

The additional difficulty arises from the challenging desymmetrization of PTCDA starting materials for the synthesis of asymmetric PDI derivatives. PDI derivative **11** cannot be obtained from PTCDA in reasonable yields using two different amines in a simultaneous or sequential addition. To circumvent this problem, the most useful method consists in a partial hydrolysis of symmetrical PDI **1** leading to a mixed imide–anhydride compound **10**, which is followed by the second imidization reaction (Scheme 2) [89,90,91]. Another route for the preparation of asymmetric PDI **12** was achieved by direct condensation of compound **2** with two aliphatic amine derivatives of similar reactivity in a stoichiometric ratio [78].

### 3.3. Perylenemonimide (PMI)-Based Building Blocks for Designing Architectures

**Scheme 3:**
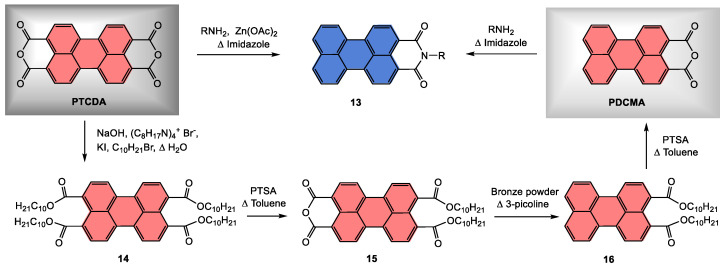
Synthesis of 3,4-perylenedicarboxylic acid monoanhydride (PDCMA) and PMI derivatives.

PMI and PDI units share the imide, ortho and bay positions on their perylene skeleton, giving them relatively close reactivity. However, an additional functionalizable position with the highly synthetically reactive peri position provides a specificity to the PMI unit. The procedure, which consists in the treatment of PTCDA with a primary amine in the presence of zinc acetate and molten imidazole, produces PMI derivative **13** in a satisfactory yield, but this compound is accompanied by perylene and PDI **1** [53]. This synthesis was improved by an efficient four-step sequence, involving reactions all in high yield and without specific purification, finally leading to 3,4-perylenedicarboxylic acid monoanhydride (PDCMA) (Scheme 3).

**Scheme 4:**
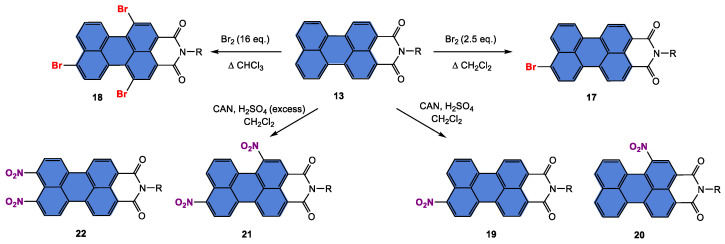
Synthesis of PMI-based building blocks.

The most important functionalization of the PMI backbone occurs firstly in the more reactive peri position and secondly in the bay positions (Scheme 4). Selective bromination affording 9-monobromoPMI **17** or 1,6,9-tribromoPMI **18** can be efficiently obtained by adjusting the solvent, bromine equivalents and reaction temperature conditions [92]. We should note the recently described procedure to perform the nitration of PMI derivatives in a more selective manner [93]. By analogy with the recent chemistry developed on nitro-PDI derivatives, the synthetic potential offered by these new compounds which are likely to react by nucleophilic substitution should be highlighted. The reaction using CAN in the presence of sulphuric acid at ambient temperature affords a mixture of major 9-mononitroPMI **19** and minor 1-mononitroPMI **20**. With an excess of reagents, 1,9-dinitroPMI and 9,10-dinitroPMI **21** and **22**, respectively, can be obtained.

**Scheme 5:**
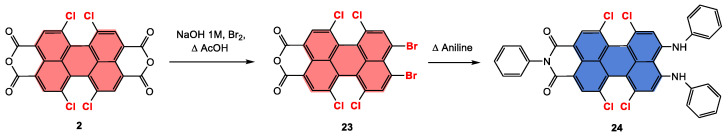
Synthesis of 9,10-dibromo-1,6,7,12-tetrachloroperylene monoanhydride building block using Hunsdiecker reaction.

To complete the range of building blocks useful for PMI functionalization, it is important to highlight 9,10-dibromo-1,6,7,12-tetrachloroperylene monoanhydride **23,** which is prepared using a facile and efficient one-step reaction from compound **2** (Scheme 5) [94]. This opens the way for nucleophilic substitution to introduce donor groups the on both the 9 and 10 peri positions, as in compound **24**, but also to consider selective substitutions in the bay (substituted by chlorine atoms) and peri (substituted by bromine atoms) positions.

In an attempt to present the very diverse PDI- and PMI-based systems recently described for applications in bioimaging and photothermal (PTT) and photodynamic (PDT) therapies in a coherent way, we have chosen to present each of these families, PDI and PMI, separately. Next, we consider the effect of substitution by taking as reference the unsubstituted PDI or PMI on the core, and thus only in the imide position. Moreover, most of the syntheses described to date use tetrachloro- and dibromo-PDI or tetrachloro-PMI derivatives as starting materials. From these building blocks, the nucleophilic substitution reaction allows the introduction of electron-donating alkoxy and amino groups with a shift in the maximum absorption approaching NIR absorption, which is favourable to the targeted applications. Our third level of differentiation concerns whether we are dealing with small molecules or polymers, bearing in mind that in the latter case, the polymer chain can be grafted onto the imide position or the bay position.

## 4. Perylenediimide (PDI)-Based Systems for Bioimaging, PTT and PDT

### 4.1. Synthesis and Applications of Unsubstituted Bay PDI Materials

#### 4.1.1. Small-Molecule-Based Systems

**Scheme 6:**
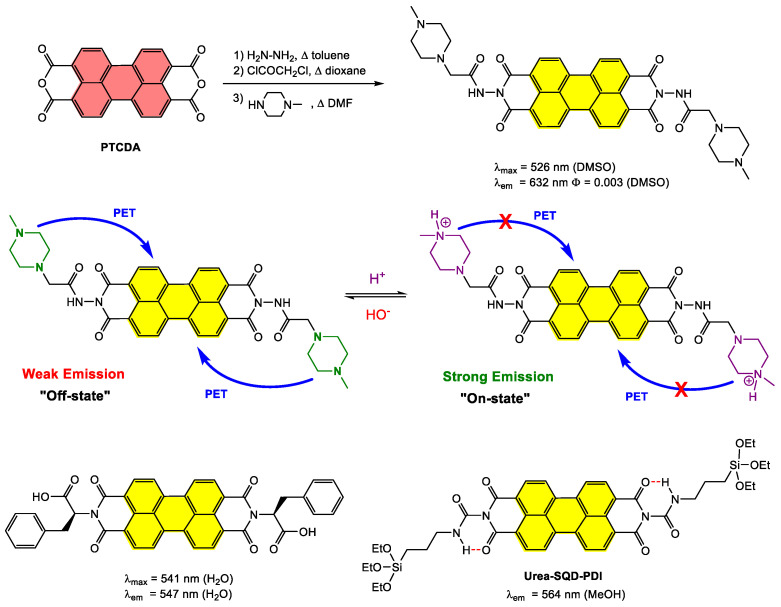
Synthesis of PDI substituted with two N-methylpiperazine groups in imide positions and pH-dependent photophysical behaviour of the probe; structures of PDI derivatives substituted in imide positions with two L-phenylalanine or N-propylurea groups.

Using PTCDA as a starting material and performing a double imidization reaction with a primary amine is the most efficient method to functionalize this motif. Therefore, very simply, a highly water-soluble PDI probe suitable for monitoring a pH variation in the physiological range was prepared (Scheme 6) [95]. This chemosensing architecture based on the fluorophore–spacer–receptor model could operate via photoinduced electron transfer (PET) from the electron-donating N-methylpiperazine groups grafted onto the imide positions in the PDI moiety. The resulting quenching of the fluorescence (off-state) was modified in the fully protonated system with amplification of the fluorescence (on-state). This PDI probe was applied to L929 cells to check their fluorescence imaging ability. The difficulty of the fluorescent dye in penetrating the cells was noted, showing that the membrane was permeable to the PDI probe only at low concentrations (1.3 μM). This probe exhibits fluorescence (on-state) in an acidic medium (pKa = 6.35 ± 0.02) and “off-state” in neutral and alkaline media. On the contrary, another PDI probe, bearing L-phenylalanine groups in imide positions, has been reported for imaging HeLa cells, with observed strong fluorescence in the pH 5.5–12 range and weak fluorescence at pH below 5.5 [96].

Silicon quantum dots (SQDs) have recently attracted increasing interest due to their superior optical properties, including wide absorption spectra, excellent stability against photobleaching and size-dependent tuneable photoluminescence (PL). A water-soluble SQD-N-propylurea-PDI assembly showed efficiency in fluorescent imaging embryonic-kidney HEK293 cells and human-bone cancerous U2OS cells. These SQD nanoparticles with a size of about 1.6 nm were constructed by heating PTCDA with 3-aminopropyl)triethoxysilane, which was previously reduced by trisodium citrate dihydrate in glycerol [97]. This **Urea-SQD-PDI** was sensitive to pH changes with a decrease in the emission intensity collected at 480 nm over a pH range of 2.6–4. The possibility of intramolecular hydrogen bonding between the hydrogen atom of the amine group and the oxygen of the diimide could make electron transfer less energetically active, and it is more likely that energy transfer is responsible for the observed quenching of the SQD emission.

**Figure 3:**
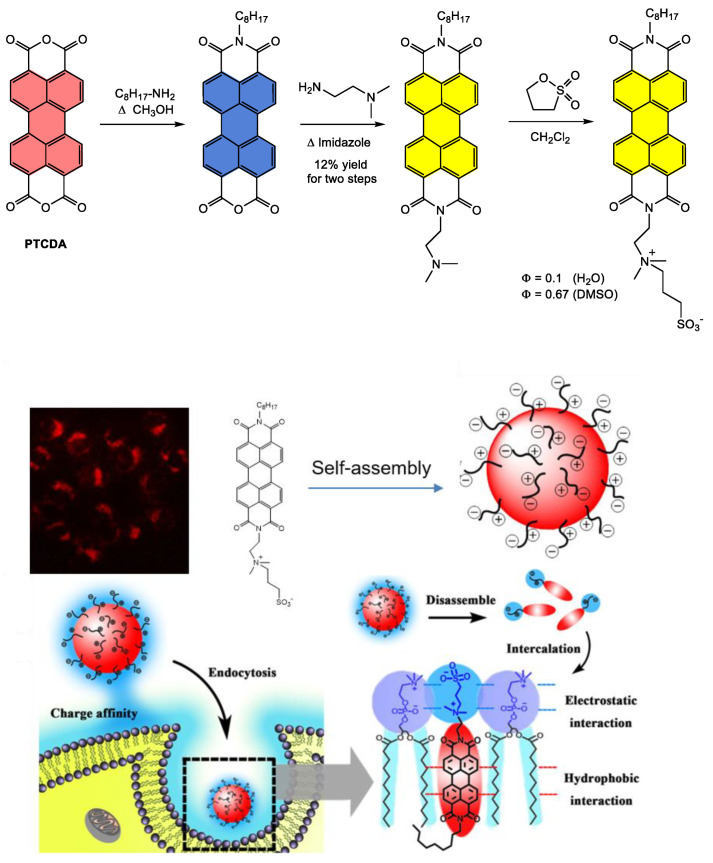
Synthesis of the amphiphilic and zwitterionic PDI derivative; illustration of self-assembly and disassembly of vesicles into cell membrane and imaging of HeLa cells. Reprinted with permission from reference [98]. Copyright 2017 American Chemical Society.

We previously stated that the imidization carried out on PTCDA using two different amines at the same time or sequentially was not a very efficient method. An asymmetric PDI derivative was designed with a hydrophobic aliphatic tail and a hydrophilic zwitterionic head (Figure 3) [98]. Indeed, using a sequential addition of the two different amines, asymmetric PDI was obtained in a 12% overall yield. Its fluorescence spectrum showed a maximum emission peak at 542 nm and a shoulder at 584 nm in aqueous solution, which could minimize the autofluorescence in bioimaging. This amphiphilic PDI derivative aggregated in water (Ф = 0.1 in water, Ф = 0.67 in DMSO) due to *π*–*π* stacking to form nanosized vesicles. These self-assemblies could be integrated into cells through interaction with the plasma cell membrane via opposite charge pairs. Then, these vesicles disassemble in the presence of dipalmitoylphosphatidylcholine (DPPC: one of the biomembrane components) micelles into single molecules to mark the inner membrane structures of cultured cells and tissues via hydrophobic interaction.

**Scheme 7:**
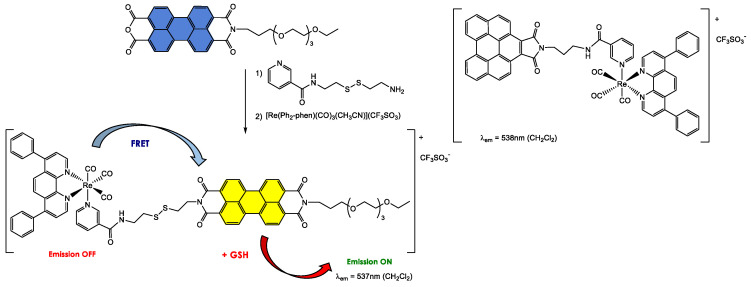
Synthesis of luminescent rhenium(I)–polypyridine complexes appended with PDI and BPMI; representation of the FRET effect occurring from the rhenium complex to the PDI acceptor.

Using the mixed imide–anhydride intermediate desymmetrization methodology, luminescent rhenium(I)–polypyridine complexes to which a PDI or a benzoperylene monoimide (BPMI) moiety was grafted through a nonconjugated disulfide linker were investigated (Scheme 7) [99]. A nonconjugated linker was defined to avoid any direct electronic communication between the rhenium complex and PDI partners. The disulfide linkage is expected to exhibit thiol sensitivity to be used as a redox trigger in drug-delivery agents. Interactions between the phosphorescent transition of metal polypyridine and a fluorescent PDI or BMPI unit could be exploited to afford new cellular reagents for bioimaging, sensing and photocytotoxic applications. Indeed, a fluorescence resonance energy transfer (FRET) effect occurring from the rhenium complex to the PDI acceptor could explain the fluorescence quenching. The best results were obtained for the BPMI-based complex which exhibited, upon irradiation at 365 nm, remarkable singlet oxygen ^1^O_2_ photosensitization efficiencies with the ability to kill HeLa cancer cells, thus confirming its excellent photocytotoxic activity as a promising photodynamic therapeutic agent.

**Figure 4:**
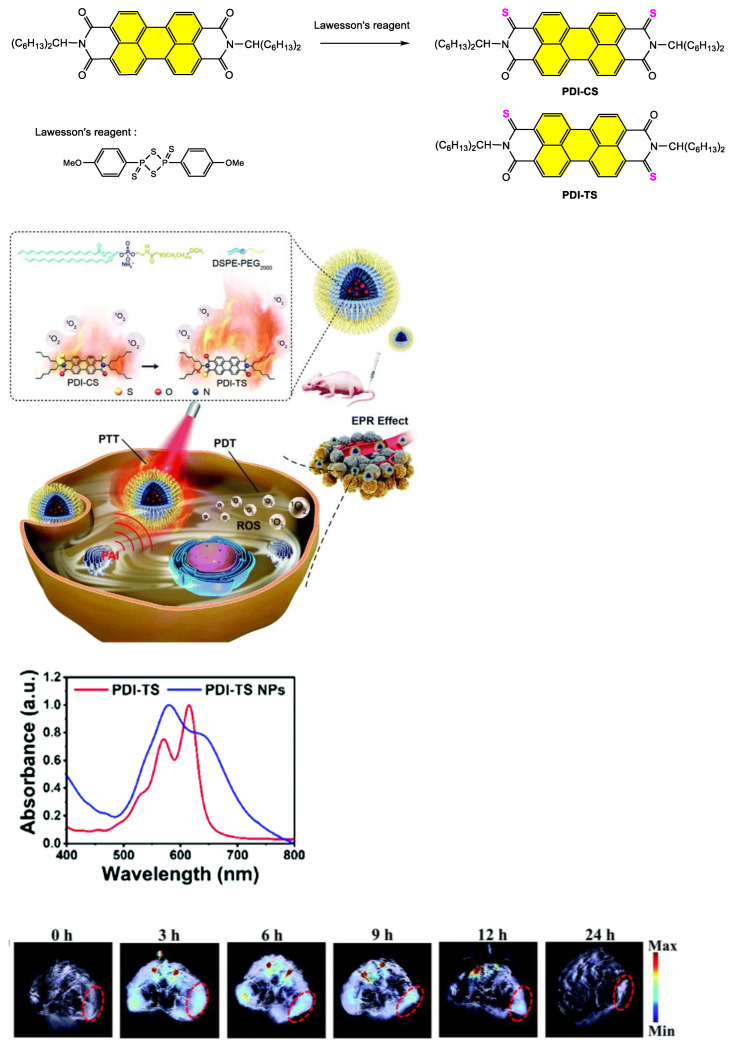
Synthesis of bis-thiono-PDIs, PDI-CS and PDI-TS; illustration of PTT and PDT in vivo using PDI-CS- and PDI-TS-based NPs; UV-Visible absorption spectra of PDI-TS in THF and NPs in deionized water; in vivo photoacoustic images in tumour-site post-injection of PDI-TS NPs. Reprinted with permission from reference [101]. Copyright 2020 Royal Society of Chemistry.

As mentioned earlier, PDT materials incorporating heavy-metal atoms can enhance the ISC process by producing triplet excitons that are effective in producing ROS via type I and/or type II processes. However, their possible toxicity poses a threat to human health. Metal-free organic materials can be more easily digested by the human body, but they rarely have the same level of ROS yield as inorganic PDT materials. Nevertheless, particular attention is now being paid to compounds incorporating a sulphur atom because of their potential biocompatibility. The slightly heavier sulphur atom compared to oxygen and its nonbonding electron pair can favour the ISC to produce triplet excitons for ROS production, thanks to the n-π* configuration as the lowest excited state in the singlet collector [100]. Original dithionated PDIs, named trans-isomer **PDI-TS** and cis-isomer **PDI-CS**, were synthesized from corresponding PDI derivatives using Lawesson’s reagent in low 5.5 and 10.9% yields, respectively (Figure 4) [101]. Such thiono-PDIs are known to exhibit a shift in their maximum absorption in the NIR region [102]. **PDI-CS** and **PDI-TS** nanoparticles (NPs) of size around 55 nm were prepared through nanoprecipitation, this NP size being compatible with target tumour tissue by the enhanced permeability retention (EPR) effect. The UV-Visible spectra of **PDI-CS** and **PDI-TS** showed absorption peaks ranging from 500 nm to 700 nm, indicating a nonaggregated form in the THF solution. However, the **PDI-TS**-based NPs in water were characterized by a weak absorption around 560 nm, which was accompanied with a new absorption peak around 660 nm, suggesting the presence of *π*–*π* interactions in these NPs. Thanks to favourable C…S interactions, photothermal depression was evidenced in A549 cells upon 660 nm light irradiation in vitro and in vivo, highlighting a higher photothermal conversion efficiency (PCE) of 58.4% for **PDI-TS** NPs to be compared with 41.6% for **PDI-CS** NPs. Notably, it was also shown that **PDI-TS** NPs could produce ROS upon 660 nm laser irradiation and have an inhibitory effect on tumour growth.

**Figure 5:**
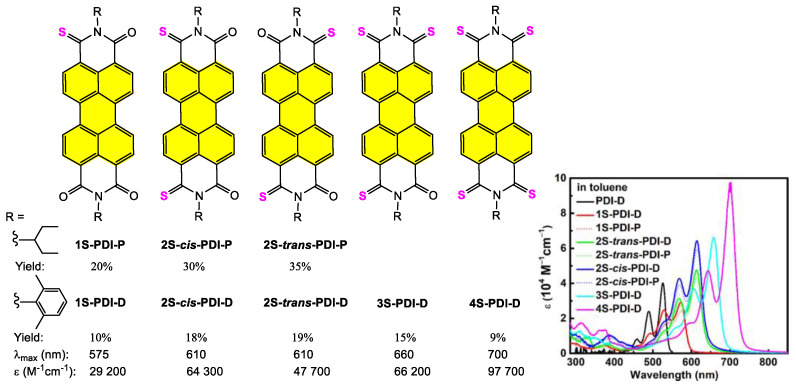
Structures of thiono-PDIs; corresponding UV-Visible spectra. Reprinted with permission from reference [100]. Copyright 2022 American Chemical Society.

This synthetic strategy has recently been applied to a series of thiono-PDI derivatives presenting one to four sulphur atoms, **1S-PDI-D**, **2S-cis-PDI-D**, **2S-trans-PDI-D**, **3S-PDI-D** and **4S-PDI-D** (Figure 5) [100]. Interestingly, the gradual introduction of several sulphur atoms shifted the maximum absorption to the NIR region with an increase in the molar-extinction coefficient. Moreover, these compounds are not emissive in common organic solvents, probably due to the fast rate of ISC which quenches the emission, but on the other hand, they showed excellent two-photon absorption.

**Scheme 8:**
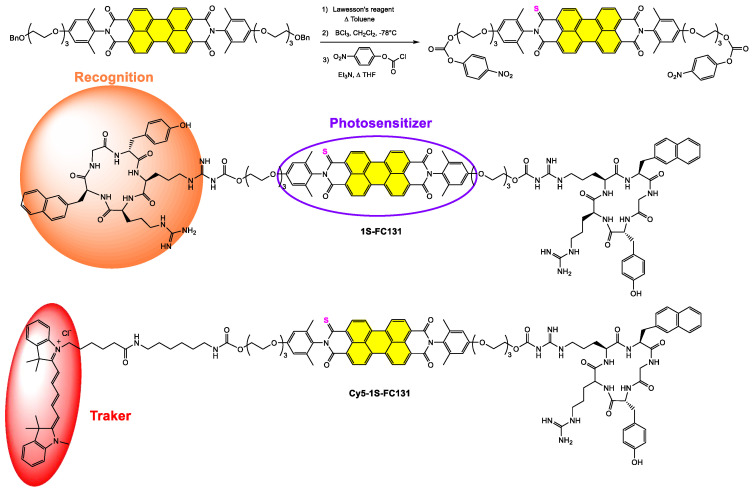
Synthesis and structures of 1S-FC131 and Cys-1S-FC131 assemblies.

A synthetic route was developed to couple **1S-PDI-D** with one peptide FC131 grafted onto each imide position, affording a **1S-FC131** system. In that case, **1S-PDI-D** acts as a photosensitizer due to its 100% ^1^O_2_ generation ability. Compound **1S-PDI-D** was also linked on one imide side with peptide **FC131**, and on the other side with cyanine5 dye, yielding a **Cy5-1SFC131** system (Scheme 8) [100]. In vitro and in vivo results confirmed that these thiono-PDI-based **1S-FC131** and **Cy5-1S-FC131** assemblies are selective PDT materials exhibiting sufficient two-photon absorption and imaging capability, and an excellent anticancer effect. In particular, both molecular systems demonstrated outstanding antitumour ability in vivo in A549 xenografted tumour mice, where **Cy5-1S-FC131** showed superiority in simultaneous fluorescence tracking and targeted PDT.

#### 4.1.2. Polymer-Based Systems

**Figure 6:**
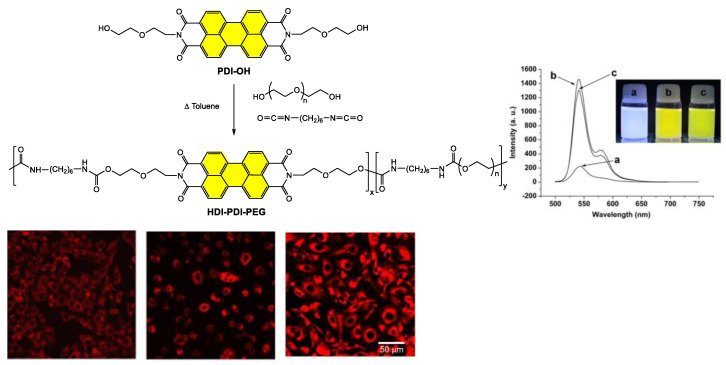
Synthesis of HDI-PDI-PEG copolymer; fluorescence spectra of (a) HDI-PDI-PEG_600_, (b) HDI-PDI-PEG_1500_ and (c) HDI-PDI-PEG_2000_ at a concentration of 0.6 mg/mL in water; confocal laser scanning microscopy images of L929 cells (**left**), macrophages (**middle**) and skov-3 cells (**right**) in a medium containing 0.125 mg/mL HDI-PDI-PEG_1500_. Reprinted with permission from reference [103]. Copyright 2016 Elsevier.

In order to solve the problem of poor solubility limiting the potential biomedicinal applications of PDI derivatives, PDI-incorporated polyurethanes **HDI-PDI-PEG** were easily prepared using a one-step condensation procedure (Figure 6) [103]. Copolymers constructed using hexamethylene diisocyanate (HDI) and poly(ethylene glycol) (PEG) showed high solubility in polar organic solvents and in water. The fluorescence in water proved to be dependent of the PEG molecular weight (HDI-PDI-PEG_600_, HDI-PDI-PEG_1500_, HDI-PDI-PEG_2000_). The highest PDI content led to the lowest fluorescence intensity due to the more favourable *π–π* stacking interactions in this case. When using **HDI-PDI-PEG_1500_**, labelled cells, including L929 cells, macrophages and skov-3 cells, emit strong red fluorescence from their cytoplasm. The isocyanate groups were further functionalized with folic acid (FA), and the fluorescence of FA-HDI-PDI-PEG_1500_ remained unchanged after folic-acid modification, thus demonstrating the great potential of using **HDI-PDI-PEG** as a universal cell-labelling agent.

**Figure 7:**
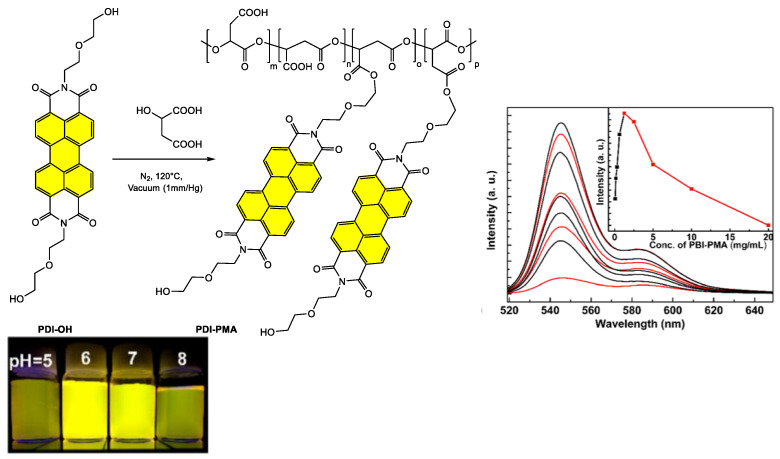
Synthesis of PDI-PMA polymer; fluorescence spectra of PDI-PMA in water; fluorescence spectra of the copolymer PDI-PMA in the buffers with various pH values (C = 1.25 mg·mL^−^^1^) with *λ*_exc_ = 488 nm. Reprinted with permission from reference [104].

Starting from this **PDI-OH** derivative, a one-step polycondensation was carried out in the presence of L-malic acid under vacuum, affording a PDI-poly(*α*,*β*-malic acid) named **PDI-PMA** of a high molecular weight around 5.8 kDa (Figure 7) [104]. This polymer introduced 3%wt **PDI-OH,** and the presence of two hydroxyl groups on the PDI starting material did not induce cross-linking in the resulting polymer. The fluorescence intensity of **PDI–PMA** in water initially increased linearly up to 1.25 mg·mL^−1^, and then decreased in the concentration range from 1.25 to 20 mg·mL^−1^, suggesting fluorescence quenching due to π–π stacking between perylene cores. Moreover, the fluorescence was shown to be pH dependent, with a maximum intensity at pH = 6–7. This polymer **PDI-PMA** displayed biocompatibility and low cytotoxicity, good water solubility and strong green fluorescence and demonstrated high potential as a cell-labelling agent using L929 and HeLa cell lines.

**Scheme 9:**
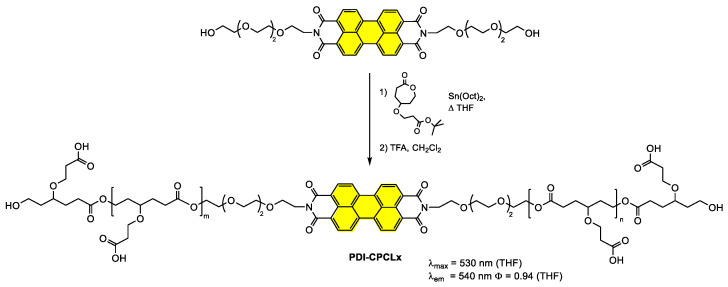
Synthesis of PDI-tagged polycaprolactone block copolymers PDI-CPCLx.

PDI derivatives are recognized for their important red-fluorescent properties associated with a high quantum yield which is suitable for imaging in biological systems [24,48,49,74]. An enzyme-responsive and biodegradable red-fluorescent PDI-tagged polycaprolactone (PCL) block copolymer named **PDI-CPCLx** was used as a nanoprobe for intracellular bioimaging in cancer and normal cells (Scheme 9). The symmetrical bis-imidization of PTCDA afforded a bis-hydroxyl functionalized PDI derivative which was used as the initiator for a ring-opening polymerization (ROP) methodology to attain a maximum of x = 40 repeating units. The deprotection of the tert-butyl esters groups gave the corresponding amphiphilic **PDI-CPCLx** block copolymers. Thanks to the self-assembly of carboxylic blocks, these copolymers exhibited nanofibrous morphology in organic solvents and spherical stable NPs with a size ~100 nm in an aqueous medium. Moreover, these NPs presented a quantum yield (Ф = 0.25–0.30), which was suitable for bioimaging applications. Cytotoxicity studies confirmed the excellent biocompatibility of **PDI-CPCLx** NPs and their accumulation in the perinuclear environment of normal and cancer cells, considering that they were taken up by the cells via the endocytosis pathway [105].

**Figure 8:**
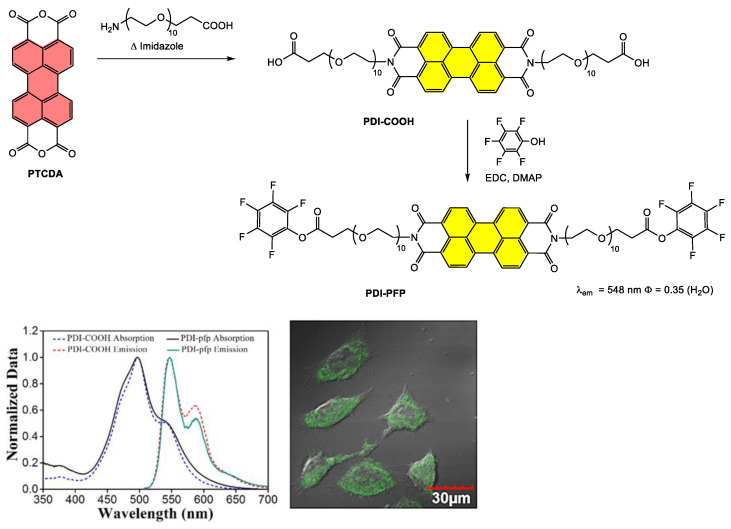
Synthesis of polymer PDI-PFP; UV-Visible and fluorescence emission spectra of PDI-COOH and PDI-PFP in aqueous solution; confocal laser scanning microscopy images of MCF-7 cells incubated with PDI-PFP (10 μm) for 30 min. Reprinted with permission from reference [107]. Copyright 2018 American Chemical Society.

Despite its well-known immunogenicity [106], the PEG polymer is still widely used as a gold standard for biomedicinal applications such as bioconjugation, drug delivery, biosensing, and imaging due to its high solubility in aqueous media. The association of PEG with fluorescent PDI derivatives was considered in the imide position (Figure 8). The **PDI-PFP** system was synthesized through imidization using H_2_N-PEG_10_-COOH; then, the corresponding free carboxylic acid was esterified under mild conditions with pentafluorophenol. This assembly was designed to offer good solubility in water due to the PEG chain, to present the active pentafluorophenyl (PFP) ester acting as the reactive group under physiological conditions and the fluorescent PDI unit. Compound **PDI-PFP** exhibited a bright green fluorescence with a maximum emission at 548 nm and an absolute fluorescence quantum yield of 0.35 in aqueous solution, which showed good potential for bioimaging. Moreover, **PDI-PFP** rapidly interacted with different types of cells, including cancer cells (MCF-7 and HeLa cells), acting as an imaging agent of the endoplasmic reticulum with a demonstrated low cell cytotoxicity [107].

**Figure 9:**
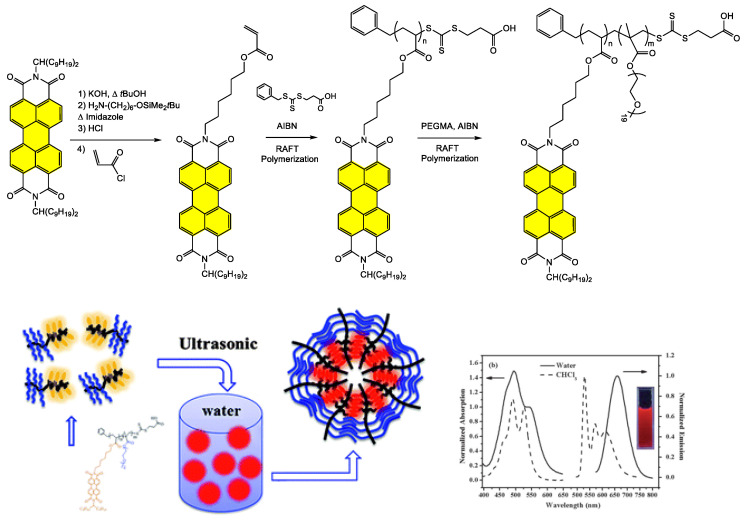
Synthesis of copolymer PPDA-b-P(PEGMA); schematic representation of the preparation of PPDA-b-P(PEGMA) PNPs; normalized absorption and emission of the PPDA-b-P(PEGMA) in water (solid line) and chloroform (dashed line). The inset shows the PNPs in water exposed to 365 nm excitation. Reprinted with permission from reference [112]. Copyright 2014 Royal Society of Chemistry.

PDI derivatives exhibit poor solubility in water and fluorescence in the visible range, which dramatically restricts their application as efficient NIR fluorescent probes. To overcome this poor-solubility drawback, the introduction of ionic groups, polyglycerol dendrons or poly(ethyleneglycol) methacrylate (PEGMA) using atom-transfer radical polymerization (ATRP) in the imide positions brought about the needed water solubility [108,109,110,111]. Considering that only a few water-soluble PDI-based probes show NIR emission, an amphiphilic diblock copolymer, poly(PDI acrylate)-block-poly(poly(ethyleneglycol) methacrylate), called **PPDA-b-P(PEGMA)**, was synthesized via the reversible addition fragmentation transfer (RAFT) polymerization method (Figure 9) [112]. An enhanced *π*–*π* stacking in the **PPDA-b-P(PEGMA)** copolymer was noted in comparison with that of the PDI acrylate (PDA) monomer. Furthermore, compared with the emission peaks at 530 and 570 nm of the PDA monomer in chloroform, a new red emission peak at 620 nm of **PPDA-b-P(PEGMA)** appeared due to the formation of *π–π* stacking in the diblock copolymer. The formation of more efficient *π–π* stacking in aqueous solution than in chloroform resulted from an aggregation behaviour and self-assembly in water. Homogeneous polymer nanoparticles (PNPs) in aqueous solution were formed with an average size around 65 nm. Cellular imaging of human pancreatic cancer cells was conducted with the obtained PNPs with a localization specifically within the cell cytoplasm, providing a new design concept of polymer to fabricate NPs with NIR emission for applications in bioimaging.

### 4.2. Synthesis and Applications of Tetrachloro Bay-Substituted PDI Materials

Most of the syntheses described to date use tetrachloro- and dibromo-PDI derivatives as starting materials. From these building blocks, nucleophilic substitution allows the introduction of electron-donating alkoxy and amino groups with a bathochromic shift in the maximum absorption, which is favourable for the targeted applications. We therefore consider the different types of substitution in the bay region, namely with chlorine atoms, alkoxy groups and, finally, amino groups.

#### 4.2.1. Small-Molecule-Based Systems

**Figure 10:**
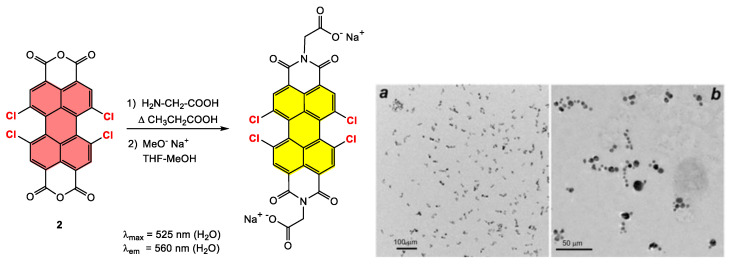
Synthesis of PDI bis-glycine carboxylate; (**a**) and (**b**) transmission electronic microscopy images of D-g-PNIPAM/Au NP hybrid macromolecules at 20 °C at two different magnifications. Reprinted with permission from reference [114]. Copyright 2022 Elsevier.

The substitution of the PDI motif in the bay positions leads to a twisting of the backbone mainly for steric hindrance reasons, with the consequence that the aggregation phenomenon is reduced. Water-soluble 1,6,7,12-tetrachloro-PDI bearing carboxylate groups in the imide positions was prepared in two steps from building block **2**. The imidization reaction using glycine in refluxing propionic acid [113] was followed with treatment by a sodium hydroxide solution (Figure 10). This PDI derivative with a maximum absorption at 525 nm in water was incorporated to reach a hybrid nanosystem composed of triple components Dextran-g-Poly(N-isopropylacrylamide)/Au nanoparticles/PDI (D-gPNIPAM/AuNPs/PDI) [114]. This system could act as a thermoresponsive fluorescent optical switch in aqueous solution in which the change in the intensity of photoluminescence was shown to result from the competing effects of plasmonic enhancement and the nonradiative FRET effect of electronic excitation energy during the thermo-induced lower critical solution temperature (LCST) transition of the D-g-PNIPAM macromolecule.

**Figure 11:**
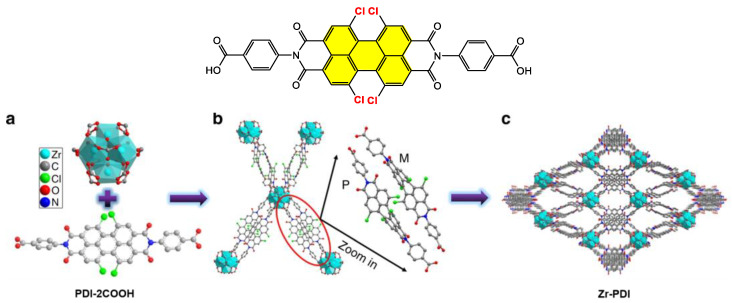
Structure of ligand PDI-2COOH; (**a**) Structures of Zr-cluster and PDI-2COOH, (**b**) connection mode of Zr-cluster, (**c**) a-Axis crystal structure of Zr-PDI. Reprinted with permission from Ref. [115]. Copyright 2019 Nature Publishing Group.

A PDI-based 3D metal–organic framework (MOF), named **Zr-PDI**, composed of a N,N′-di-(4-benzoic acid)-1,6,7,12-tetrachloro-PDI (PDI-2COOH) ligand and Zr_6_(μ3-O)4(μ3-OH)_4_ clusters has been reported (Figure 11). Characterized by the high stability of its radical anions, this framework showed high NIR PCE (η = 52.3%). This efficient and simple method to reach stable radical anions affords a promising material for PTT [115].

**Figure 12:**
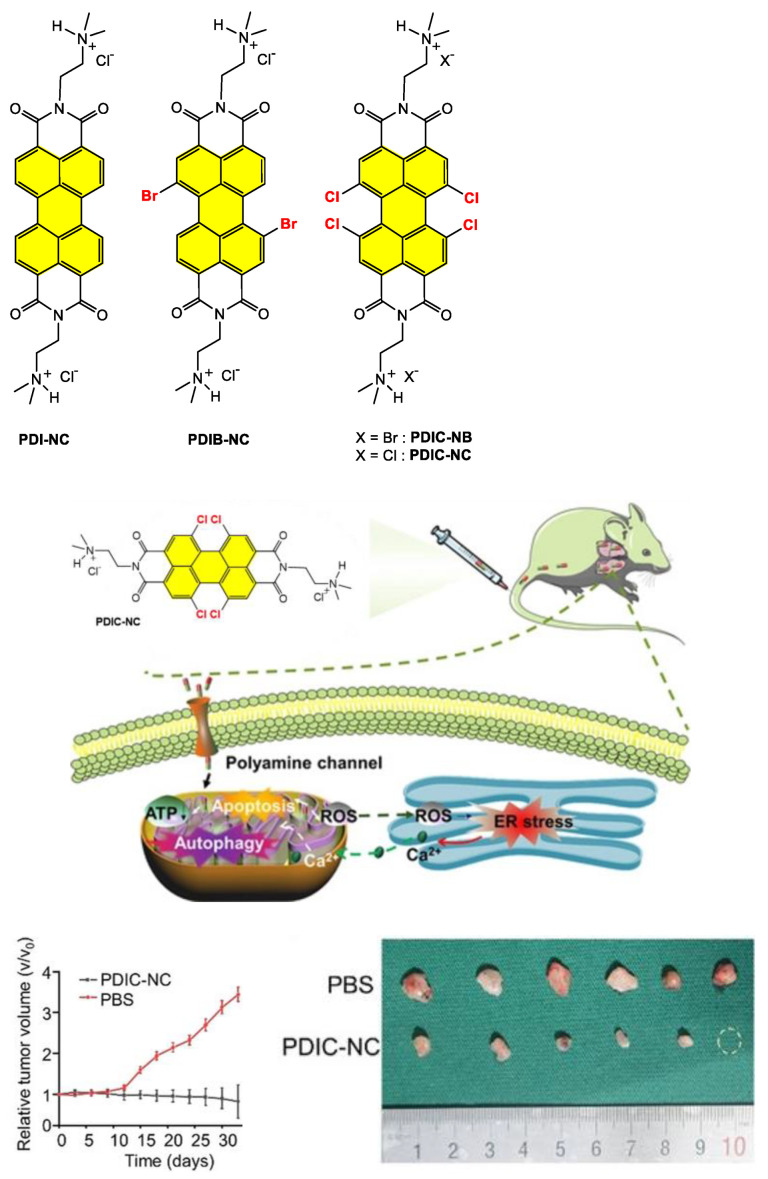
Structures of PDI-NC, PDIB-NC, PDIC-NB and PDIC-NC molecules; in vivo antitumour activity of PDIC-NC on a subcutaneous xenotransplanted tumour with the tumour tissue images in PBS and PDIC-NC treatment mice and relative tumour volume. Adapted with permission from reference [116]. Copyright 2021 Wiley-VCH GmbH.

The use of anti-cancer drugs is a trade-off between therapy and side effects. Therefore, it is necessary to design drugs for organ-specific delivery. It was recently shown that PDI derivatives can be delivered specifically to the lung and target mitochondria to act as an inhibitor of cellular respiration. The influence of the degree of substitution over the bay region of zero (**PDI-NC**), two (**PDIB-NC**) or four (**PDIC-NC**) substituents was studied. After intravenous injection, only a lung-specific distribution in an A549 xenografted tumour, A549 and H446 metastasis tumour and orthotopic tumour was achieved for the **PDIC-NC** derivative, by comparing with phosphate-buffered saline (PBS)-treated cells. This result suggests that lung-specific distribution is strongly associated with the twisted perylene skeleton and less related to the nature of the anion (Figure 12) [116].

#### 4.2.2. Polymer-Based Systems

##### Grafting the Chain Polymer in the Imide Position

**Scheme 10:**
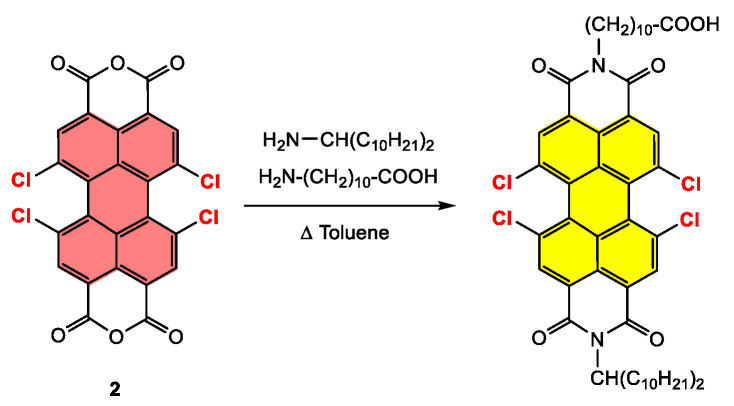
Preparation of the PDI precursor for synthesis of PLGA-PEI polymer.

An asymmetric 1,6,7,12-tetrachloro-PDI derivative was linked to polyethylenimine-g-poly(lactide-co-glycolide)-g-polyethylenimine (**PLGA-PEI**) polymer to obtain fluorescent multifunctional polymer. Then, a peptide sequence was grafted using the free carboxylic group to attain multifunctional micelles labelled with fluorescent PDI (Scheme 10) [117]. These micelles exhibited enhanced photobleaching stability, and the fluorescent images of cellular uptake show bright red emission. These PDI-labelled micelles have shown the ability to condense DNA-forming micelle–DNA complexes with great potential for the gene-delivery process.

##### Grafting the Chain Polymer in the Bay Position

**Figure 13:**
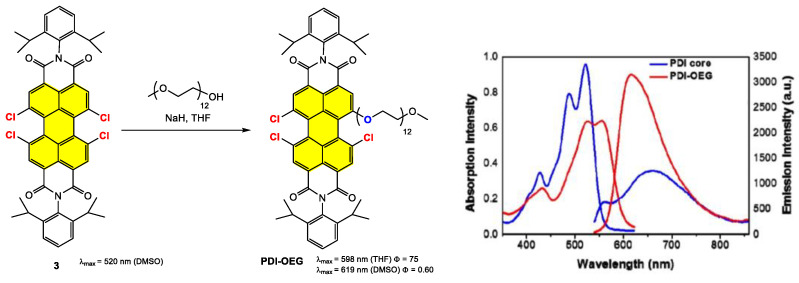
Synthesis of PDI-OEG; UV-Visible and fluorescence spectra of PDI **3** and PDI-OEG in PBS solution. Reprinted with permission from reference [118]. Copyright 2016 Optica Publishing Group.

Selective nucleophilic substitution with a single chain of oligoethylene glycol (OEG) chain on 1,6,7,12-tetrachloro-PDI derivative **3** was described in a 45% yield (Figure 13) [118]. The UV-Visible absorption and fluorescence spectra of PDI **3** and **PDI-OEG** were measured in PBS. While the fluorescence of PDI **3** exhibited intense emission with a maximum at 669 nm in PBS, **PDI-OEG** was found to be nonfluorescent in PBS. The hypothesis of fluorescence quenching of **PDI-OEG** in PBS due to micelle generation was discarded. This phenomenon is explained by the increased interactions between the charged phosphate groups in buffer solution and the OEG moieties in **PDI-OEG** leading to fluorescence quenching. Fluorescence-imaging studies were carried out using multiple cancer cell lines, and ex vivo studies in mouse models showed that **PDI-OEG** specifically targeted the liver and lung organs, demonstrating that this fluorophore could be a good example of a stable and biocompatible red-emitting small molecule for bioimaging.

### 4.3. Synthesis and Applications of Dialkoxy and Tetraalkoxy Bay-Substituted PDI Materials

#### 4.3.1. Small-Molecule-Based Systems

**Figure 14:**
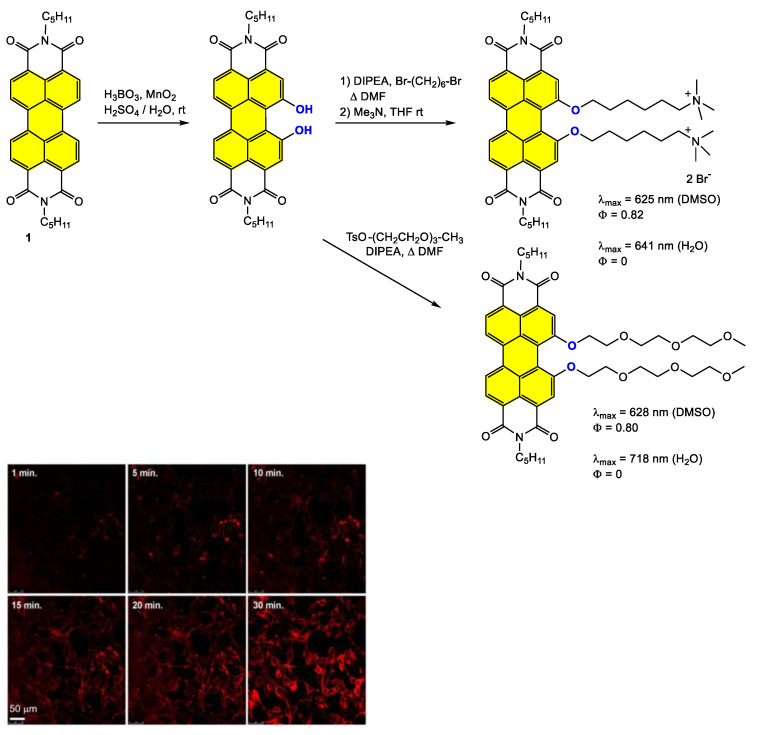
Synthesis of 1,12-dialkoxy PDI derivatives; confocal laser scanning microscopy images of HeLa cells in the first 30 min after administration of a 5 µM solution of PDI Gemini-type surfactant. Reprinted with permission from reference [119]. Copyright 2018 Wiley-VCH GmbH.

The original 1,12-dihydroxylated PDI derivative was synthesized by a treatment of boric acid and manganese oxide in acidic medium in a quantitative yield (Figure 14) [119]. Then, this starting material was O-dialkylated with a triethyleneglycol chain or an alkyl chain bearing a final-ammonium salt. The latter material can be considered as a Gemini surfactant [120] bearing two hydrophilic head groups, two hydrophobic groups and a rigid fluorescent PDI core. These 1,12-dialkoxy PDI derivatives exhibit maximum absorption around 630 nm in DMSO, whereas a bathochromic shift was observed in water. It is important to note the fluorescence quenching in water, suggesting the formation of aggregates. The cationic PDI Gemini-type surfactant acted as an off–on fluorescent probe since it formed nonfluorescent self-assembled particles in water (“off-state”), with appealing high fluorescence upon incorporation into lipidic bilayers (“on-state”), showing a significant Stokes shift, with potential for use as a bioimaging probe.

#### 4.3.2. Polymer-Based Systems

**Figure 15:**
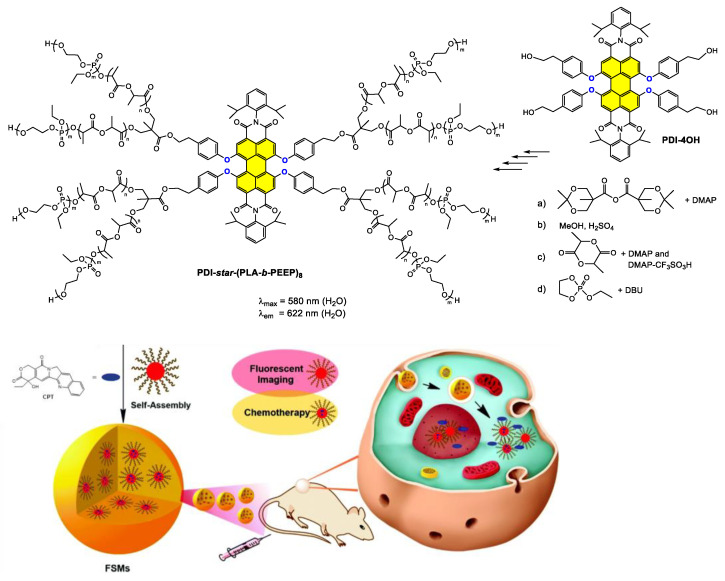
Synthesis of PDI-star-(PLA-b-PEEP)_8_; formation of nanomicelles and fluorescence-imaging-guided intracellular drug release. Scheme adapted from reference [121]. Copyright 2016 Royal Society of Chemistry.

In order to graft the chain polymer onto the bay position, a smart fluorescent PDI-based drug-delivery system (DDS) composed of a block copolymer poly(D,L-lactide)-b-poly(ethyl ethylene phosphate), named **PDI-star-(PLA-b-PEEP)_8_**, was developed as a biodegradable unimolecular micelle. This can self-assemble into fluorescent supramolecular micelles (FSMs) with controllable sizes in an aqueous solution (Figure 15) [121]. The key step of the synthesis is based on a ROP reaction using D,L-lactide and the initiator PDI bearing eight alcohol groups, in the presence of 4-dimethylaminopyridine (DMAP) and DMAP-CF_3_SO_3_H as the catalysts. A second ROP of ethyl ethylene phosphate (EEP) using the PDI-star-PLA_8_ with 1,8-diazabicyclo[5.4.0]undec-7-ene (DBU) as catalyst afforded **PDI-star-(PLA-b-PEEP)** in an overall 35% yield from **PDI-4OH**.

**Figure 16:**
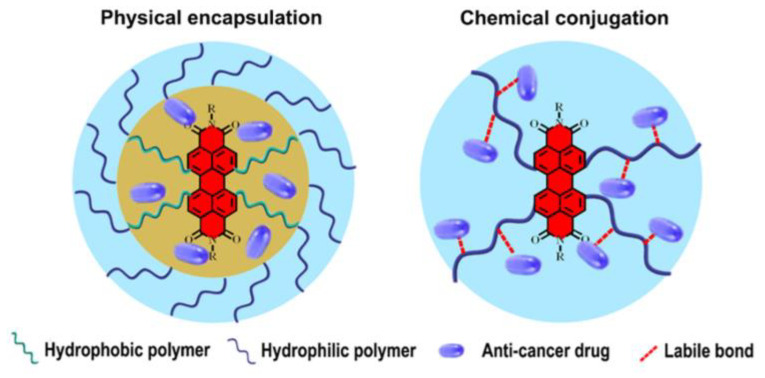
Physical and chemical loading of chemodrug with PDI-cored polymers. Reprinted with permission from reference [74]. Copyright 2019 American Chemical Society.

It was shown that FSMs can play a dual role: (i) the hydrophobic drug camptothecin (CPT) can be loaded within FSMs through physical encapsulation (Figure 16). NPs of a size of 60 nm exhibited an EPR effect with accumulation in the tumour region. The chemodrug effect was demonstrated by its subsequent pH-responsive release; (ii) FSMs can act as a fluorescent probe for cell labelling thanks to their maximum absorption at 580 nm and emission at 622 nm, which can minimize the interference from cell autofluorescence. Micelle FSMs are easily endocytosed by cancer cells, and a better therapeutic effect of FSMs after CPT encapsulation was observed in in vitro and in vivo tumour growth when compared with the free-CPT drug. This phenomenon could be explained by a physical encapsulation of the chemodrug into the hydrophobic cavity of the PDI-based amphiphilic polymer or by chemical conjugation to pending functional groups of the polymer through cleavage bond (Figure 16).

**Figure 17:**
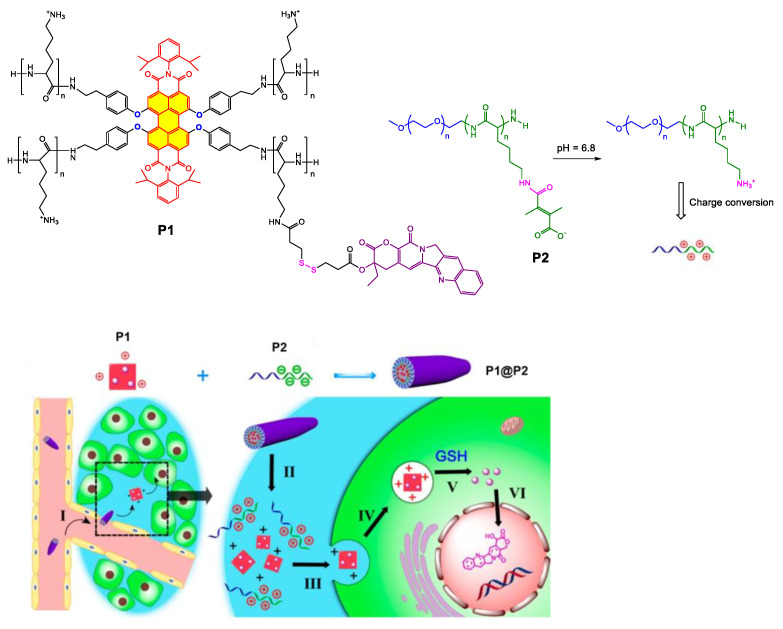
Chemical structures of P1, P2 and P1@P2 complex; illustration of the CPT-delivery process using the P1@P2 supramolecular complex. Adapted and reprinted with permission from reference [122]. Copyright 2017 American Chemical Society.

As an illustration, a supramolecular DDS (SDDS) was designed with two oppositely charged building blocks. The first one has a fluorescent star polycation (**P1)** linked with CPT through a reduction-responsive bond, and the second one consists in an anionic copolymer (**P2**). The interaction between **P1** and **P2** led to complex **P1@P2** which is stable in blood and accumulates at a tumour site through an EPR effect (I) (Figure 17) [122]. In the tumour extracellular microenvironment, cleavage at pH = 6.8 of the amide bond led to a charge conversion and the disassembly of complex **P1@P2** (II). The enhanced cellular uptake (III) induced the prodrug to be released through a “proton sponge” effect (IV). Due to the disulfide-bond cleavage in the presence of overexpressed glutathione (GSH) within the tumour microenvironment, CPT was subsequently released from the cationic prodrug (V) to enter the cell nucleus (VI) and show its cytotoxity in the apoptosis of cancer cells. The complex displayed better tumour-inhibition efficiency when compared with that of pure CPT, indicating the potential of this drug-delivery concept.

### 4.4. Synthesis and Applications of Amino-PDI Bay-Substituted Materials

#### 4.4.1. Small-Molecule-Based Systems

**Figure 18:**
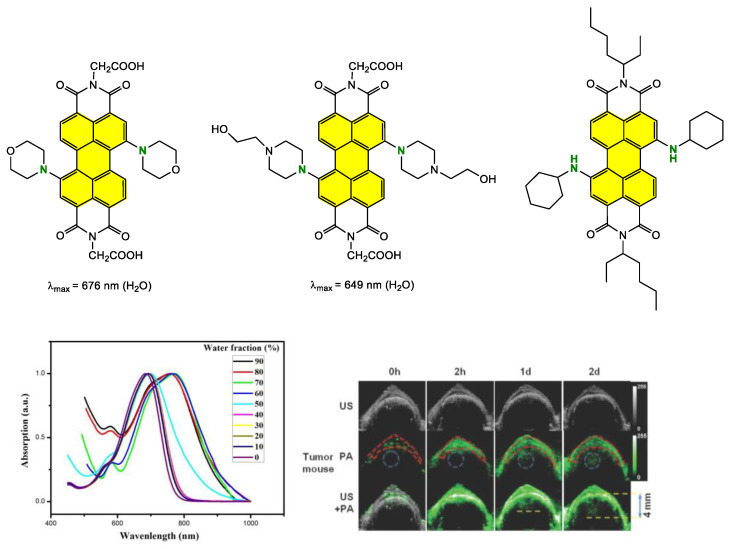
Structures of 1,7-bis-morpholino, 1,7-bis-piperidino and 1,7-bis-cyclohexylamino PDI; normalized absorption spectra of 1,7-bis-cyclohexylamino PDI in THF/water mixtures with different volume ratios; photoacoustic imaging and ultrasound imaging in the skull and tumour region in brain-bearing mice of 1,7-bis-cyclohexylamino PDI NPs. The dashes are used to delineate (red) and measure (yellow) the size of the tumour. Reprinted with permission from Ref. [124]. Copyright 2015 Wiley-VCH GmbH.

The fine and controlled tuning of the optical properties of PDIs is of great interest for engineering fluorophores. To respond to this demand, the functionalization with amine in the bay position through nucleophilic substitution is a common approach. The amino group drastically changes the optoelectronic properties of PDI, with a red shift in the maximum absorption and a decrease in the fluorescence quantum yield. This is a consequence of a charge transfer between the amino group and the PDI unit, owing to the HOMO located on the amine and the LUMO on the PDI. The presence of an amino group in the bay PDI position was demonstrated to facilitate the ISC involved in the photodynamic process. Moreover, an increase in the PCE of up to 43% in compounds bearing amino groups was shown in the bay region. In fact, upon light excitation, these compounds undergo strong internal conversion and vibrational relaxation which transform the absorbed light into heat through nonradiative decay, resulting in the destruction of cancer tissues when temperature exceeds 45 °C [74]. A wide variety of such PDI derivatives were synthesized by direct nucleophilic substitution from the dibromo precursor using the appropriate amino compound, i.e., morpholine [123], piperidine [123] or cyclohexylamine [124] derivative (Figure 18). Starting from the 1,7-biscyclohexylamino PDI derivative, water-soluble and highly stable PDI NPs were obtained through enveloping the hydrophobic PDI with amphiphilic DSPE-mPEG_5000_. These PDI-based NPs with a particle size around 50 nm exhibited NIR absorption of around 700 nm in THF with an impressive extinction coefficient of 2 × 10^8^ M^−^^1^·cm^−^^1^. Increasing the content of water in the THF mixture, an additional red-shifted absorption peak of PDI at around 780 nm was formed, indicating the typical *π*–*π* intermolecular aggregation of PDI after the addition of water. Furthermore, a weak fluorescence of PDI was found in THF, while no emission existed in the THF/water mixture, indicating that PDI aggregation could efficiently produce fluorescence quenching. These PDI NPs were successfully used to realize a PAI of deep orthotopic brain tumours in mouse models, showing that they could act as photoacoustic (PA) contrast agent for in vivo deep-brain tumours with a simple detection using the EPR effect.

**Figure 19:**
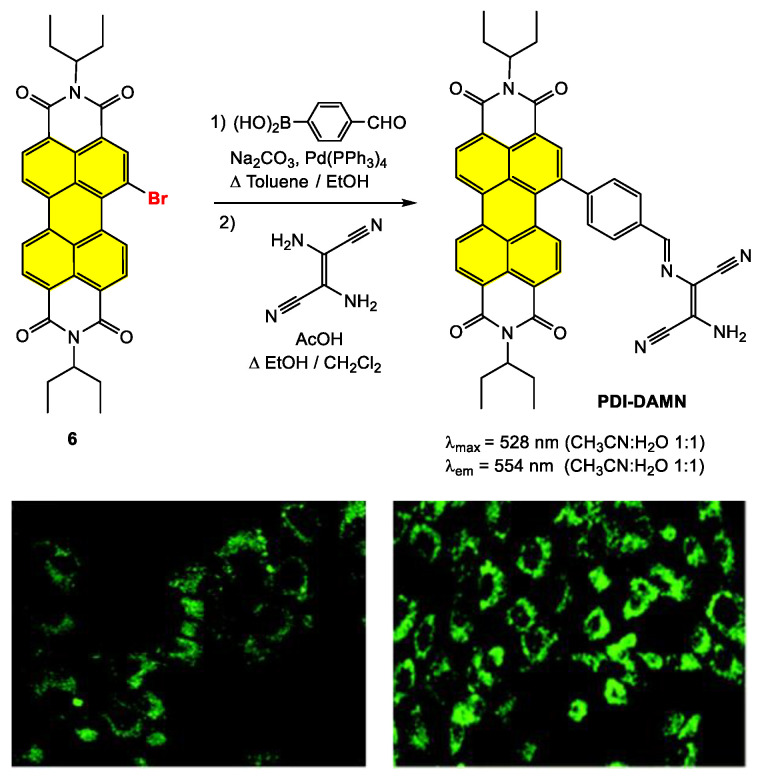
Structure of PDI-DAMN; fluorescent imaging of exogenous ClO^−^ anion in MG-63 cells after incubation with PDI–DAMN (10 mM) then with ClO^−^ anion (left); fluorescent imaging of endogenous ClO^−^ anion in MG-63 cells after incubation with lipopolysaccharides (LPS) and phorbol myristate (PMA), then with **PDI–DAMN**. Images have been taken at magnification 40×. Reprinted with permission from Ref. [125]. Copyright 2019 Royal Society of Chemistry.

A fluorescent water-soluble probe **PDI-DAMN** dyad containing a diaminomaleonitrile (DAMN) moiety in the bay region was synthesized for the detection and bioimaging of hypochlorite (ClO^−^) anion in cells [125]. The DAMN motif has not been grafted directly onto the PDI core and, therefore, the presence of the spacer does not allow the benefit of the absorption bathochromic shift. Starting from bromo-PDI derivative **6**, a pallado-catalysed Suzuki–Miyaura reaction using 4-formylphenyl boronic acid was followed by the condensation with diaminomaleonitrile (Figure 19). **PDI-DAMN** self-assembled as nanofibers with diameters in the range of 100–200 nm in CH_3_CN: H_2_O (1:1). The addition of ClO^−^ anion into PDI–DAMN resulted in the disintegration of nanofibers into flake-like aggregates of a smaller size (50–80 nm). The application of **PDI-DAMN** for the bioimaging of both exogenous and endogenous ClO^-^ anion in MG-63 cells with good biocompatibility was also demonstrated.

**Scheme 11:**
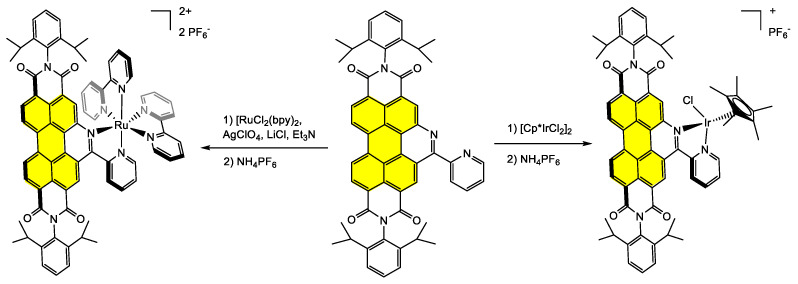
Synthesis of ruthenium(II) and platinum(III) complex azabenzannulated PDI-based derivative.

Whereas PDI monomers and dimers functionalized in the bay region with Pt (II)-acetylide linkage have previously been shown to give rise to a triplet-excited state leading to a reasonable ^1^O_2_ quantum yield [126,127,128], original ruthenium(II) and platinum(III) complexes of an azabenzannulated PDI-based derivative were recently synthesized (Scheme 11) [129]. The potential of these transition-metal complexes for photodynamic applications was investigated. It was clearly demonstrated that the production of ^1^O_2_ using the Ir^III^ complex upon light irradiation at 420 nm was favoured (85% yield) compared to the lower production of the Ru^II^ complex (29% yield). The phototoxicity towards cancer cells was consistent with this ^1^O_2_ generation, and the Ir^III^ complex showed a remarkable phototoxicity. Nevertheless, it should be noted that its very low solubility in biological media is the main drawback to be addressed [130].

#### 4.4.2. Polymer-Based Systems

##### Grafting the Chain Polymer in the Imide Position

**Figure 20:**
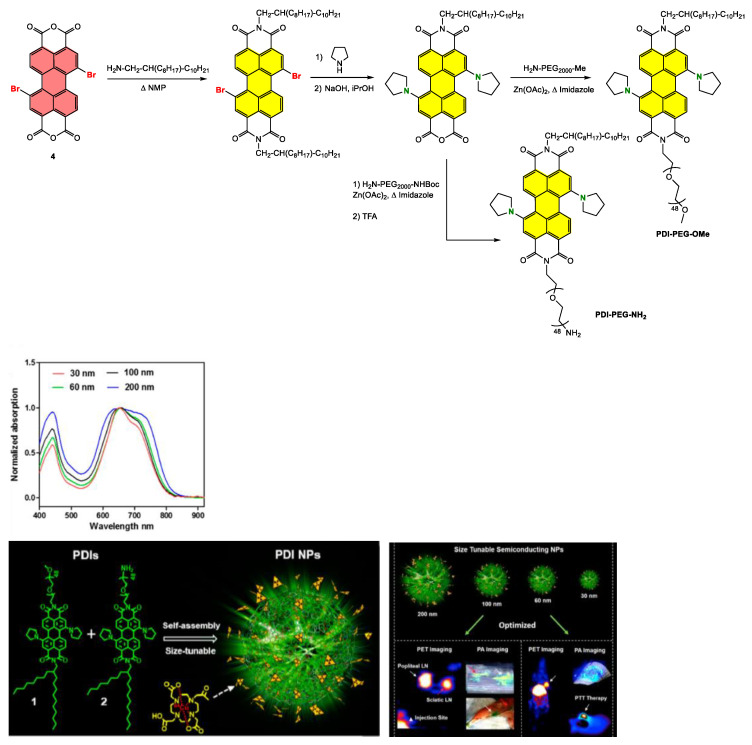
Synthesis of asymmetric amphiphilic PDI derivatives; normalized UV-Visible spectra of different-sized PDI NPs in water; formation of the PDI NPs and their potential applications. Reprinted with permission from reference [131]. Copyright 2017 American Chemical Society.

Semiconducting PDIs are of great interest thanks to their NIR-light absorption and excellent biocompatibility to be used as PAI agents. Amphiphilic PDI derivatives were synthesized using a desymmetrization strategy in the imide positions and were further self-assembled, producing a series of PDI NPs of different sizes, from 30 to 200 nm (Figure 20). The absorption peak at 700 nm became broader, in agreement with the formation of H-aggregates, when increasing the size of the PDI NPs. These NPs were labelled with radionuclide [^64^Cu] for positron emission tomography imaging. It was demonstrated by intravenous injection that the NPs with a diameter of around 60 nm had the best properties for tumour imaging and photothermal cancer therapy due to the maximization of tumour-accumulation efficiency [131].

**Figure 21:**
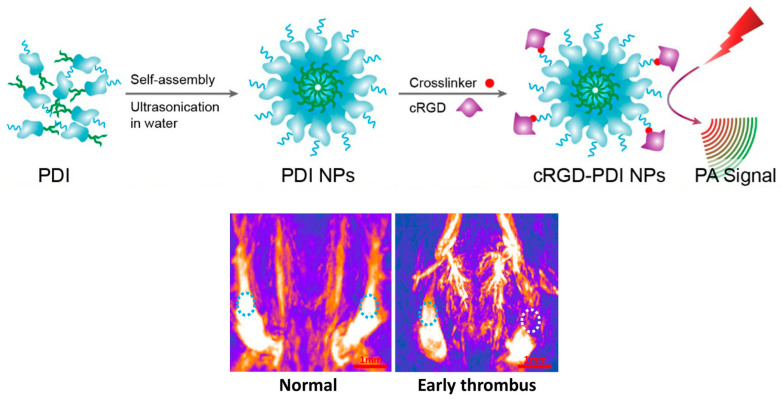
Schematic illustration of the preparation of cRGD-PDI NPs; PAI showed normal jugular veins (in blue circle) and jugular veins with early thrombus (in white circle). (**Left**): normal mice. (**Right**): thrombus model. Reprinted with permission from Ref. [132]. Copyright 2017 American Chemical Society.

Using the amphiphilic **PDI-PEG-NH_2_** macromolecule described above, **cRGD-PDI** NPs were prepared by association with a cyclic Arg-Gly-Asp (cRGD) peptide playing the role of a PA contrast agent. These **cRGD-PDI** NPs exhibited special properties with high PA intensity, high biocompatibility and an affinity for the GPIIb/IIIa receptor for the accurate diagnosis of early thrombus (Figure 21) [132].

**Figure 22:**
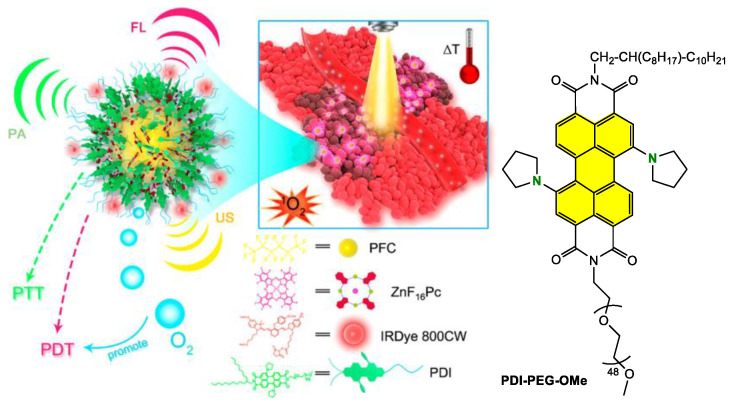
Photoacoustic nanodroplet PS-PDI-PAnD for in vivo photothermal and photodynamic therapies. Reprinted with permission from reference [133]. Copyright 2018 American Chemical Society.

Ultrasound imaging (USI) is one of the imaging techniques frequently used in clinics owing to its noninvasion, nonionization and relatively low cost. Moreover, the combination of PA and USI offers high-spatial-resolution images with deep-tissue penetration. Recently, phase-changeable perfluorocarbon (PFC) nanodroplets have been investigated as an alternative ultrasound contrast agent due to their excellent tumour vascular permeability. Thus, a photoacoustic (PA) nanodroplet, **PS-PDI-PAnD**, was developed for stabilizing low-boiling-point PFC droplets, with **PDI-PEG-OMe** playing the role of photoabsorber and PA agent, but also with ZnF_16_Pc acting as the photosensitizer (Figure 22) [133]. The PDI derivative described above was designed to exhibit amphiphilic properties thanks to the long alkyl chains, and to stabilize the shell of the nanodroplets due to their strong π−π stacking. Upon irradiation of the **PS-PDI-PAnD** molecules with a 671 nm laser light (*λ*_max_ of ZnF_16_Pc), the PDI shell can efficiently convert light energy into heat, triggering the liquid-to-gas phase conversion of the PFC core for contrast-enhanced USI to induce a photothermal effect on cancer cells. On the other hand, the encapsulated ZnF_16_Pc photosensitizer can transfer light energy to the oxygen, resulting in the generation of cytotoxic ^1^O_2_ for enhancing the PDT effect.

**Figure 23:**
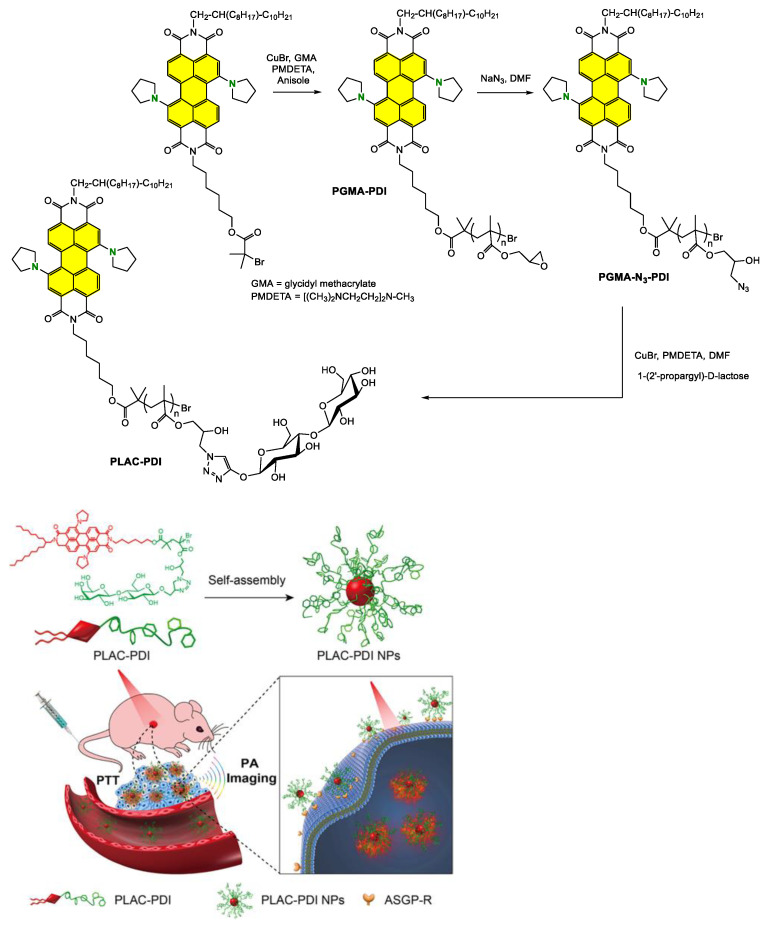
Synthesis of PLAC-PDI and illustration of the preparation of PLAC−PDI NPs and PAI/PTT applications to HepG2 tumours. Reprinted with permission from Ref. [134]. Copyright 2017 American Chemical Society.

The ideal nanoagent for cancer therapy applications with functions of PAI and PTT should present high absorption in the NIR region, high PCE, excellent photostability and biocompatibility and high accumulation in tumour tissue. Considering that PDI proved to be an efficient PAI contrast for lightening the brain tumour and early thrombus in living mice due to its strong light absorption in the NIR region and excellent photostability, the next step consisted in the development of coated glycopolymers with a tumour-targeting ability for efficient PAI and PTT [134]. An amphiphilic poly(lactose)-modified PDI (**PLAC−PDI**) was synthesized via sequential ATRP and Huisgen-type click reaction (Figure 23). The glycoplolymers self-assembled in an aqueous solution to form **PLAC−PDI** NPs which exhibited good water solubility, high density of grafted lactose, low cytotoxicity and an excellent PCE of 42%. Furthermore, **PLAC−PDI** NPs presented in vitro and in vivo a specific targeting ability and enhanced PTT efficacy to HepG2 tumours.

**Figure 24:**
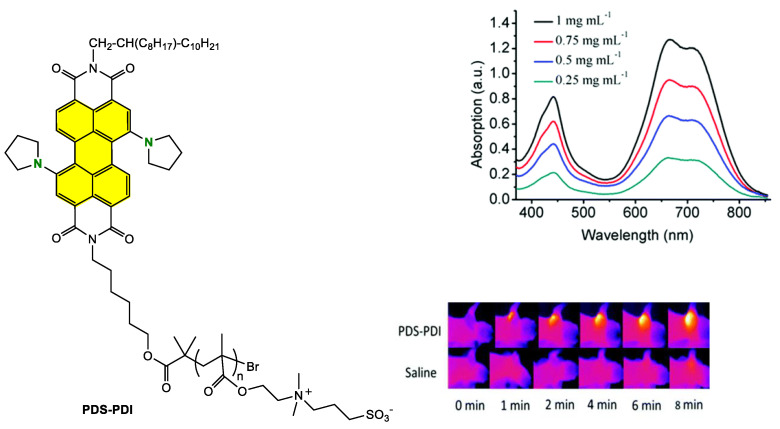
Structure of PDS-PDI; UV-Visible spectra of PDS-PDI in water at different concentrations; photothermal images of tumour tissue samples from MDA-MB-231 tumour-bearing mice intravenously injected with PDS-PDI (2 mg mL^−1^, 100 μL) or saline upon 660 nm laser (0.5 W cm^−2^) irradiation. Reprinted with permission from Ref. [135]. Copyright 2018 Royal Society of Chemistry.

Using a similar synthetic strategy and ATRP, a zwitterionic polymer **PDS-PDI** was designed for PAI-guided PDT and PTT (Figure 24) [135]. This polymer exhibited high PCE (η ≈ 40%) and efficient singlet oxygen quantum yield (Φ ≈ 16.7%) under 660 nm laser irradiation. Acting as a contrast agent for PAI and allowing for the real-time monitoring of tumour sites, the **PDS-PDI** polymer showed in vitro and in vivo effective tumour killing ability under 660 nm laser irradiation.

**Figure 25:**
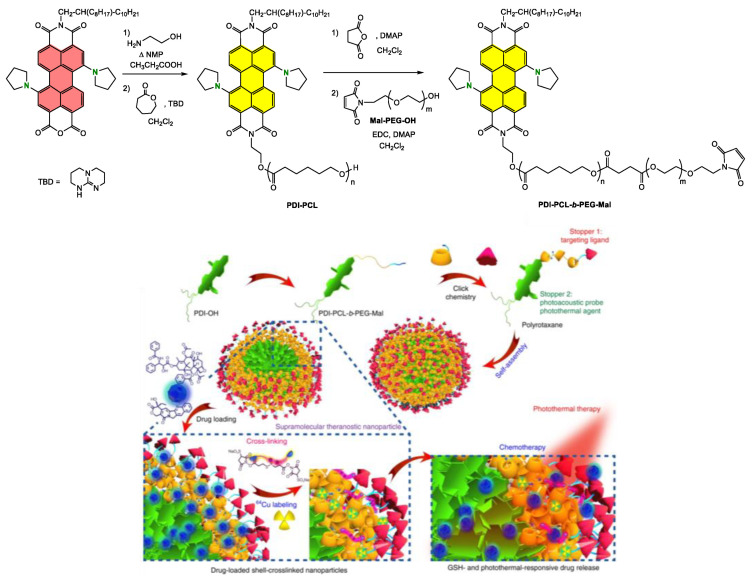
Synthesis of PDI-PCL-b-PEG-Mal; illustration of the preparation of drug-loaded SCNPs and dual-responsive drug release using polyrotaxane. Reprinted with permission from Ref. [136]. Copyright 2018 Nature Publishing Group.

This methodology of desymmetrization was used to synthesize theranostic shell-crosslinked nanoparticles (SCNPs) using a β-cyclodextrin (CD)-based polyrotaxane (PDI-PCL-b-PEG-RGD⊃β-CD-NH_2_) (Figure 25) [136]. The objective of this strategy was to develop supramolecular nanomedecine by employing polyrotaxane as a theranostic platform. In this case, drug-loaded SCNPs can prevent premature leakage of the drug and allow precisely controllable release, thereby improving the maximum tolerated dose. The polyrotaxane (PDI-PCL-b-PEG-RGD⊃β-CD-NH_2_) was synthesized starting from the mixed imide–anhydride compound. First, **PDI-PCL** was obtained in a two-step process through imidization using ethanolamine, then a ROP reaction using *ε*-caprolactone, with this alcohol functionality on PDI as an initiator. Further reaction with succinic anhydride and then esterification with Mal-PEG-OH produced the diblock copolymeric axle **PDI-PCL-b-PEG-Mal**. A polypseudorotaxane inclusion complex (PDI-PCL-b-PEG-Mal⊃β-CD-NH_2_) was formed by the host–guest interactions between PDI-PCL-b-PEG-Mal and β-CD-NH_2_, which self-assembled into NPs in an aqueous solution. Remarkably, drug-loaded SCNPs have been shown in vivo to completely eliminate subcutaneous tumours without recurrence after a single-dose injection, combining chemotherapy and PTT. These supramolecular nanodrugs also showed excellent antitumour performance against orthotopic breast cancer and prevent lung metastasis with negligible systemic toxicity.

**Figure 26:**
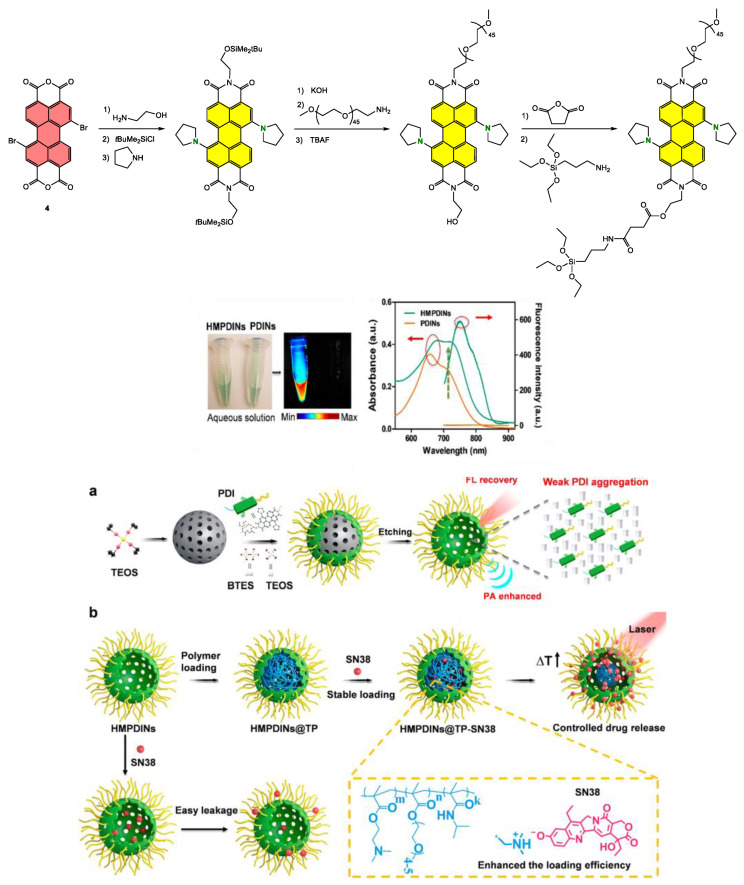
Synthetic strategy for the PEG_2000_-PDI-silane; PBS solutions of HMPDINs and PDINs and their fluorescence-imaging signal (**left**) with their UV–Visible and fluorescence-emission spectra (**right**); (**a**) synthetic route of HMPDINs and the mechanism for the recovered fluorescence and enhanced photoacoustic imaging properties, (**b**) scheme of the polymerization within HMPDINs for photocontrolled drug release. Reprinted with permission from reference [137]. Copyright 2019 American Chemical Society.

Phototheranostics refers to advanced photonics-mediated theranostic methods for cancer therapy and includes imaging-guided photothermal or photodynamic chemotherapy, particularly for the purpose of developing DDS and controlled drug-delivery release. In this field, phototheranostic hollow mesoporous nanoparticles prepared by hybridization of the PDI derivative PEG_2000_-PDI-silane within the hollow mesoporous organosilica framework were investigated [137]. Organic self-assembled PDI nanoparticles (PDINs) and the corresponding PDI-organosilica platform (HMPDINs) were prepared in water. Their comparison could demonstrate the enhanced phototheranostic properties achieved through the growth of PDI within the silica framework. The aqueous solutions of HMPDINs and PDINs exhibited a green colour with a maximum absorption of around 700 nm. However, the fluorescence of PDI in HMPDINs showed a 211.5-fold intensity enhancement compared to that of the PDINs. Consequently, the amplified fluorescence and PA signals of the silica nanoparticles, HMPDINs, can be used for PAI applications. Furthermore, the framework-hybridized PDI could generate heat under NIR-laser irradiation to trigger the deformation of the thermosensitive polymer (TP) for controlled drug release of the hydrophobic drug SN38 in the tumour region (Figure 26).

**Figure 27:**
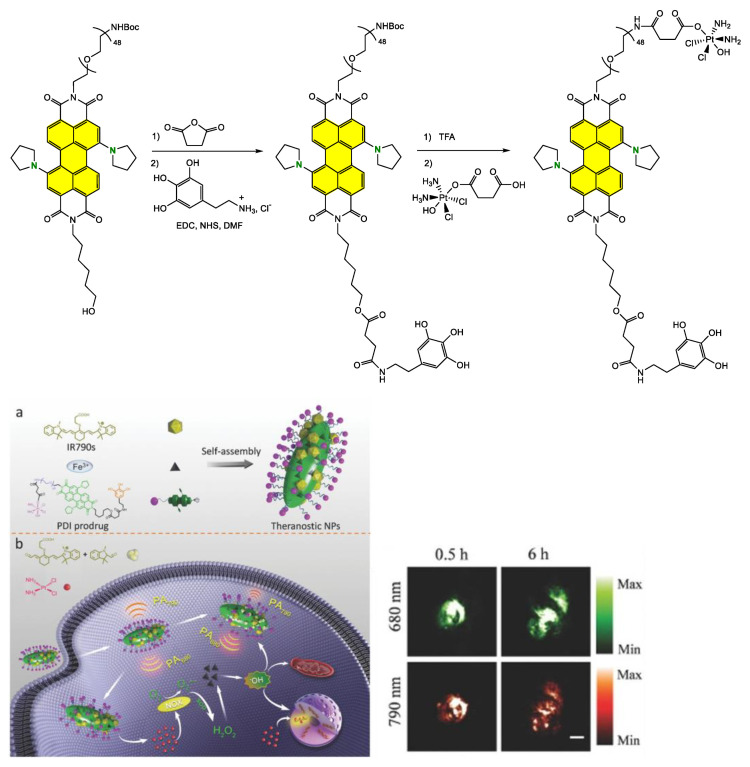
Synthesis of PDI prodrug; (**a**) illustration of the preparation of theranostics by self-assembly of PDI prodrug and IR790s triggered by Fe^3+^, (**b**) mechanism of cisplatin release; PA images of the tumour with local administration of PDI–IR790s NPs. Reprinted with permission from Ref. [138]. Copyright 2018 Wiley-VCH GmbH.

With the aim of enhancing ROS generation and facilitating the real-time observation of anticancer chemotherapy effect by ratiometric PA imaging, the original asymmetric theranostic NPs (PDI–IR790s–Fe/Pt) were constructed by self-assembly of the PDI-based cisplatin prodrug (PDI prodrug), IR790s and chelated ferric ions. The synthetic strategy followed the one described above, using 2-aminohexan-1-ol instead of ethanolamine to perform the imide substitution, and protecting the PEG-NHBoc group in the other imide position (Figure 27) [138]. The polyphenol group was introduced to coordinate with ferric ions, thus directing the self-assembly of the PDI prodrug. On the other hand, the PEG chain promoted water solubility, and a cisplatin prodrug was further conjugated to the deprotected end of the PEG chain. The presence of two pyrrolidinyl groups in the bay position provided strong NIR absorption at 680 nm, making this assembly an excellent PA probe. In the tumour microenvironment, cisplatin is released in the presence of reductive species such as glutathione, triggering the conversion of oxygen to superoxide radical (O_2_^•−^), finally leading to the formation of H_2_O_2_. These characteristics allow the theranostic nanoplatform to track ROS generation and cancer-therapy effect by ratiometric PA imaging under excitation wavelengths of 680 and 790 nm.

**Figure 28:**
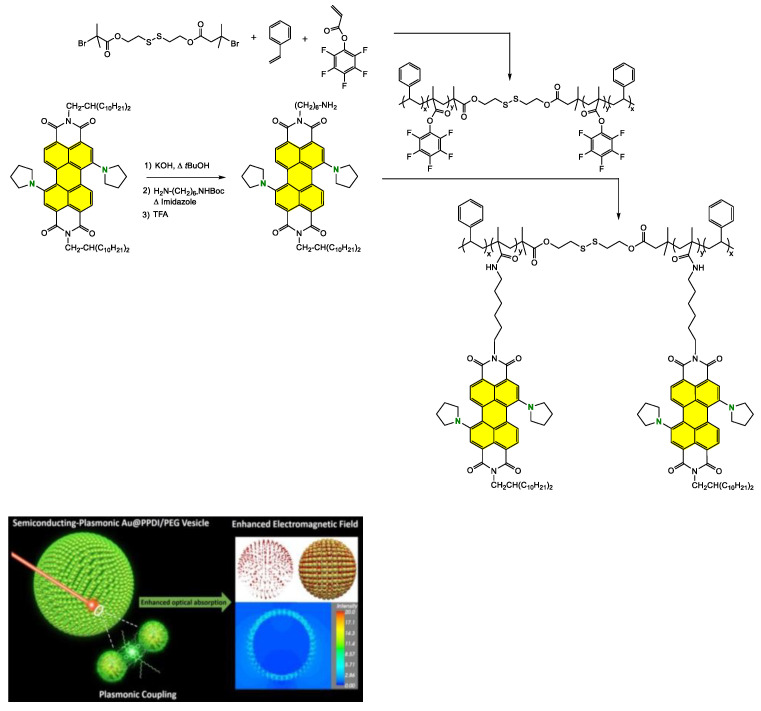
Synthetic scheme of polymer SS-PS-PPDI; illustration of the semiconducting-plasmonic vesicle of gold nanoparticle coated with PPDI and PEG. Reprinted with permission from reference [139]. Copyright 2017 Ivyspring.

While PDI small molecules and polymers have been studied previously as photoacoustic (PA) and photothermal agents [112], the use of semiconducting polymer–metal nanoparticle hybrid materials to enhance PA signals had not been explored until recently. A novel semiconducting-plasmonic Au@PPDI/PEG nanovesicle was fabricated by the self-assembly of semiconducting poly(PDI) (**PPDI**) and PEG-tethered gold nanoparticles (Figure 28) [139]. This nonconjugated polymer containing pendant PDI units was synthesized using a post-polymerization modification method. For grafting PDI units, a copolymer precursor was initially prepared with styrene and poly(pentafluorophenyl acrylate) (**PPFPMA**) acting as an activated ester by ATRP with 2,2’-dithiobis [1-(2-bromo-2-methylpropionyloxy)]ethane (DTBE) as an initiator. Gold nanoparticles (AuNPs) were obtained thanks to the disulfide functionality, which can form covalent Au-S bonds to conjugate with the AuNP surface. Characterized by a strong NIR absorption, the plasmonic coupling of the AuNPs in the vesicle enhanced the light-absorption efficiency and PA signal of PPDI. In vivo imaging and therapeutic evaluation demonstrated that the hybrid vesicle with enhanced photothermal efficiency can act as an excellent probe for high-resolution tumour imaging and cancer theranostics.

##### Grafting the Chain Polymer in the Bay Position

**Figure 29:**
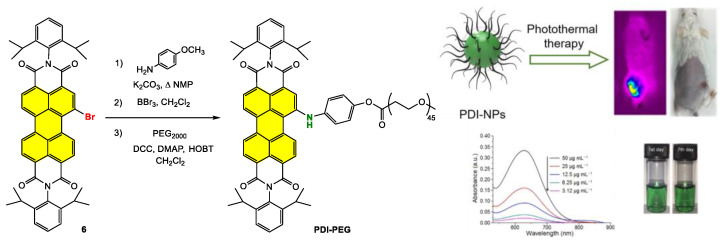
Synthesis of PDI-PEG; representation of PDI-PEG NPs; UV-Visible absorption spectrum of PDI-PEG. Reprinted with permission from reference [140]. Copyright 2018 Elsevier.

An example of the chain polymer introduction on a bay region was using bromo-PDI derivative **6** as starting material, which was submitted to a three-step sequence. A nucleophilic substitution with p-anisidine was followed by the deprotection of the methoxy group, and finally, Steglich-type esterification with PEG_2000_ possessing an active carboxylic terminal group (Figure 29) [140]. The presence of the amino group grafted on the PDI core bathochromically shifted the maximum absorption between 600 and 700 nm. The incorporation of PEG made **PDI-PEG** highly soluble in water, and corresponding NPs with a size of 55 nm exhibited quite low cytotoxicity. It was demonstrated that these amphiphilic **PDI-PEG** nanoparticles can serve as stable photothermal agents for in vivo efficient photothermal cancer therapy thanks to a high PCE of up to 43%.

**Figure 30:**
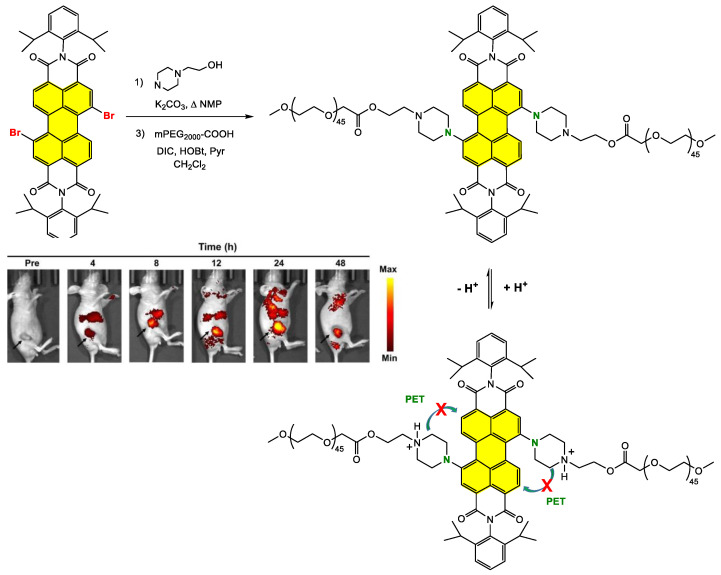
Synthesis of 1,7-bis-piperazino PDI with PEG terminal groups; mechanism of PET quenching by protonation in acidic medium; in vivo NIRF imaging of the tumour-gearing mouse after intravenous injection of PDI NPs. Reprinted with permission from reference [141]. Copyright 2020 Ivyspring.

Still using amino substituents in the bay region for attaining NIR absorption, two pH-responsive piperazine units were grafted in the 1,7-positions with terminal PEGs as side chains to reach a multifunctional phototheranostic agent (Figure 30). The obtained chromophore self-assembled into NPs in an aqueous solution, exhibiting great near-infrared fluorescence (NIRF) emission and a high PCE of 45.3% in an acidic microenvironment. Importantly, the protonation in acidic medium of piperazine rings blocks the photoinduced electron transfer (PET) process, leading to an increase in NIR emission of PDI at 760 nm, which is associated with PTT. These PDI NPs were used as organic probes for NIR fluorescence/photoacoustic/infrared thermal trimodality imaging to accurately detect the tumour [141]. Furthermore, after the injection of PDI-NPs and irradiation with a 660 nm laser, severe tumour cell necrosis damage was observed, meaning PDI NPs offer a new chromophore for developing phototheranostic cancer therapy.

**Figure 31:**
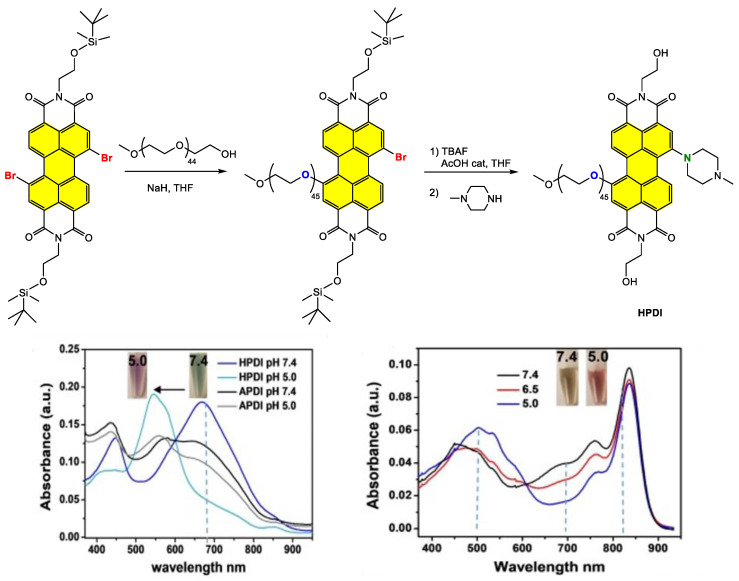

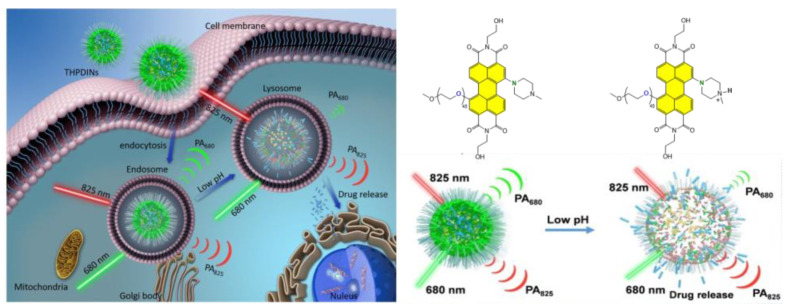
Synthesis of pH-sensitive HPDI; UV-Visible absorption spectra variation in HPDINs and APDINs at pH = 7.4 and 5.0 (**left**); UV-Visible absorption spectra of THPDINs (**right**); illustration of the THPDINs mechanism: upon internationalization, low pH environment induces a protonation reaction of HPDI, achieving pH imaging and DOX release. Reprinted with permission from reference [143]. Copyright 2019 Ivyspring.

As seen here in the previous examples, the PDI material was used successfully for PA imaging to detect tumours, thrombi and lymph nodes [124,131,132,133]. With the electron pair on the nitrogen atom acting as a donor towards the PDI acceptor, a shift in the PDI absorption from 530 nm to 680 nm is observed, thus providing an excellent contrast for PA applications. To introduce a compromise on the strength of the donor character towards the PDI unit, and with the idea to slightly blue-shift the absorption [142], asymmetric pH-sensitive nanotheranostic agents called **HPDI** and **APDI** were designed (Figure 31) [143]. The PEG and N-methylpiperazine groups were introduced by successive nucleophilic substitutions in the 1,7-positions of the PDI core, but the synthetic yields are not specified. Examples of bay-position desymmetrization are very rare and generally lead to very low yields, starting from the dibromo-PDI derivative. It should be noted that one methodology has recently reported that selectivity is more attractive starting from the dinitro-PDI analogue [85,144]. The analogous **APDI** derivative is substituted with an octyl chain in the imide positions, and the synthesis of **HPDI** and **APDI** started from the common building block dibromoperylene dianhydride **4**. The nanoagent **HPDI** was shown to be the ideal platform for PA imaging and drug-release monitoring. Indeed, with decreasing pH, **HPDI** was protonated, and absorption peaks shifted from 825 nm at pH = 7.4 to 680 nm at pH = 5.0. Consequently, a hydrophilic structural modification was promoted, accompanied with encapsulated DOX release and PA signal changes. The theranostic platform THPDINs was prepared by a nanoprecipitation method using the pH indicator **HPDI**, a pH-non-sensitive hydrophobic dye (IR825) and an anticancer drug, doxorubicin (DOX). This platform was demonstrated to inhibit the tumour growth thanks to the pH control of the DOX release in living mice, and monitored by real-time ratiometric PA imaging.

## 5. Perylenemonoimide (PMI)-Based Systems for Bioimaging and PDT

The solubility of PMI derivatives in organic solvents can be significantly increased by substitution in the imide position using an amine with long or branched alkyl chains or an aniline sterically hindered in its ortho positions acting as excellent aggregation blockers. On the contrary, the substitution of the bay positions offers the possibility to modify both the solubility and electronic properties. The distinct peri position allows nucleophilic substitution as well as the introduction of donors, such as the pyrrolidinyl group [145], or acceptor moieties and thus provides the impressive tailoring of the photophysical properties of PMIs. However, only a very limited number of water-soluble PMI derivatives have been described to date [48]. This remains the sine qua non condition for the development of biological and medicinal applications, as well as for applications in fluorescence imaging [54].

### 5.1. Synthesis and Applications of Unsubstituted Bay PMI Materials

**Figure 32:**
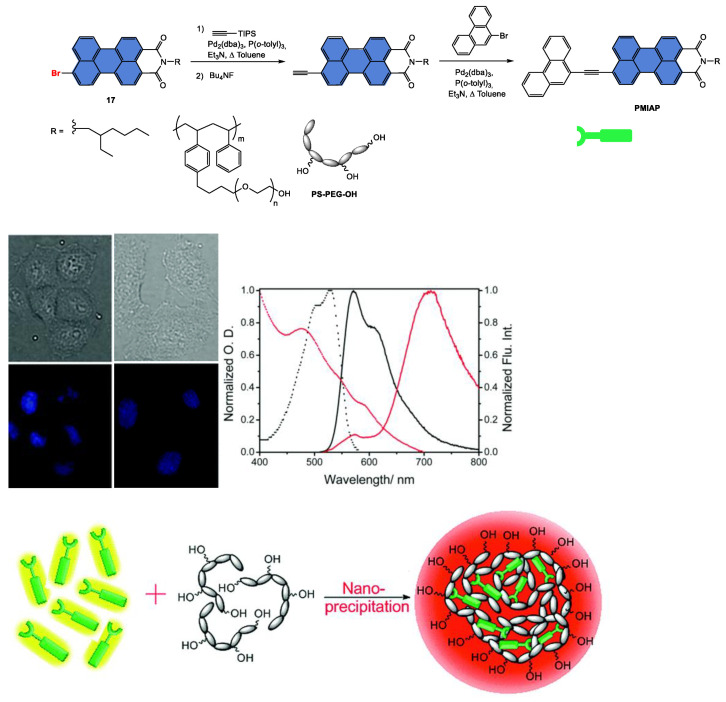
Synthetic scheme of PMIAP; UV-Visible absorption and emission spectra of PMIAP in THF (black) and upon doping inside the PNP (red); schematic representation of PMIAP-doped PNP via a nanoprecipitation method. Reprinted with permission from reference [146]. Copyright 2018 Royal Society of Chemistry.

A water-soluble NIR-emitting live-cell-imaging probe was developed by doping a hydrophobic PMI derivative called **PMIAP** in FA-conjugated PNPs (Figure 32) [146]. The synthesis of **PMIAP** was conducted using two successive Sonogashira coupling reactions, starting from the 9-bromoPMI **17** compound [147]. While **PMIAP** showed a maximum absorption at 530 nm with a shoulder at 500 nm in solution (THF, DMSO, DMF), the appearance of a broad spectrum was observed with an increase in water content (0–50%). The emission spectrum of **PMIAP** also became unstructured and was bathochromatically shifted as a result of **PMIAP** aggregate formation upon the addition of water. The emission colour upon UV-light (365 nm) illumination also showed a visible change from yellow to red with the increasing water content. The synthesis of PNPs was carried out using amphiphilic copolymer PS-PEG-OH-possessing hydroxyl groups at the periphery, which was doped with **PMIAP** and functionalized with an FA derivative. The main strategy was to achieve aggregation-induced emission (AIE) with a minimal concentration of **PMIAP** and make a water-soluble NIR-emitting fluorescent probe with a large Stokes shift. These PNPs were employed to distinguish NIR emission between folate-receptor-positive (HeLa) and -negative (MCF-7) cancer cells.

**Scheme 12:**
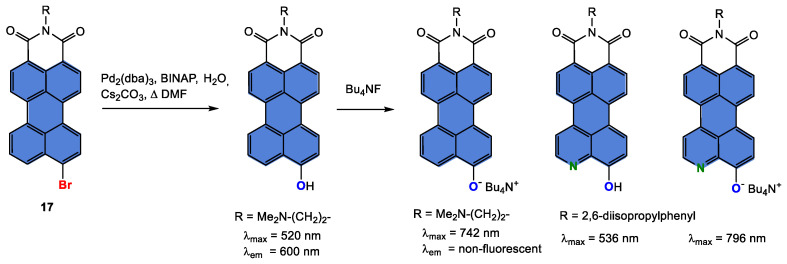
Synthesis of 9-hydroxy-PMI and its alcoholate anion.

To achieve absorption in the NIR region using the PMI backbone, the increase in conjugation appears to be a challenging task. Because of the strong electron-withdrawing property of the imide group, the introduction of an electron-donating group, such as an amino group [148], in the PMI peri position proved to be the most efficient approach. The resulting push–pull effect introduced in the electronic structure can thus induce a favourable intramolecular charge transfer to realize red-shifted absorption. The introduction of a hydroxy group in the peri position or the development of aza-PMI recently demonstrated the potential of these dyes with emission in the NIR region (Scheme 12) [149,150].

**Figure 33:**
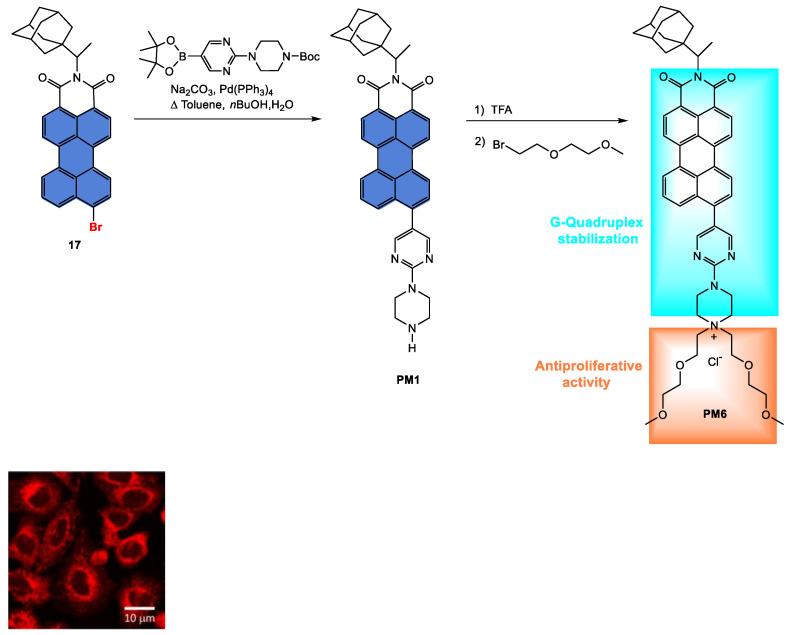
Synthesis of PM1 and PM6 derivatives presenting G-quadruplex stabilization and antiproliferase activity with a red emission in mitochondria. Reprinted with permission from reference [151]. Copyright 2021 Elsevier.

Aza-heterocycles piperazine-pyrimidine were introduced in the PMI peri position using a Suzuki–Miyaura coupling. After the deprotection of the tert-butyloxycarbonyl (Boc) group, the derivative **PM1** was bispegylated to reach water-soluble cationic compound **PM6** (Figure 33) [151]. The latter was characterized by a dual nature with the presence of a polar piperazinium group with highly hydrophilic diethylene glycol methyl ether pendant groups on one side and, on the other side, a nonpolar bulky 1-adamantyl group, suitable for hydrophobic interactions. This assembly showed a G-quadruplex binding ability and an antiproliferative activity. Interestingly, it was noted that **PM1** preferentially stabilized the parallel RNA G4 and the DNA human telomeric G4 (hTelo), while **PM6** preferred the human telomeric G4 (hTelo). Moreover, it was shown that the fluorescence of **PM6** significantly increased in the presence of G4s and was the most cytotoxic compound because of its accumulation, mainly in the mitochondria.

**Figure 34:**
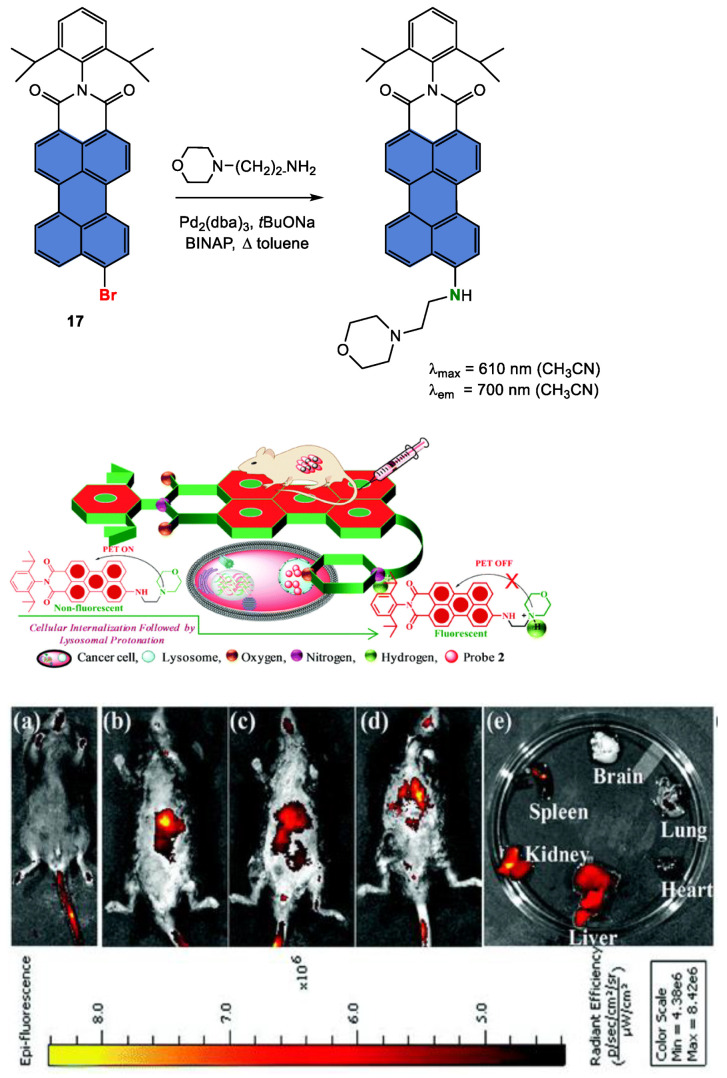
Synthesis and in vivo NIR imaging of PMI derivative in female C57BL/6J mice: (**a**) after 0 h, (**b**) 8 h, (**c**) 16 h, (**d**) 24 h, (**e**) ex vivo images of different organs after 24 h. Reprinted with permission from reference [152]. Copyright 2019 Royal Society of Chemistry.

With the introduction of a (2-morpholino)ethan-1-amino group in the peri position using a Buchwald–Hartwig amination, a PMI-based lysosome-specific NIR fluorescent probe was described (Figure 34) [152]. This compound exhibited an intense absorption band at 610 nm and an emission maximum red-shifted at about 700 nm in acetonitrile. A bathochromic shift with increased emission intensity was observed on increasing the solvent polarity, this phenomenon being attributed to the suppression of the PET from the morpholine to the PMI unit. When recorded in a mixture of CH_3_CN/H_2_O, an increase in the percentage of water resulted in a bathochromic shift in the emission maximum to around 725 nm, accompanied with a gradual decrease in the emission intensity because of the formation of aggregates. This photostable NIR probe proved to be highly specific for lyposome imaging in both fixed and live MCF7 cells. It was exploited to discriminate cancer cells over normal cells in vitro, and it was successfully used for in vivo deep-tissue imaging of a C57BL/6J mouse model.

### 5.2. Synthesis and Applications of Substituted Bay and/or Peri PMI Materials

**Scheme 13:**
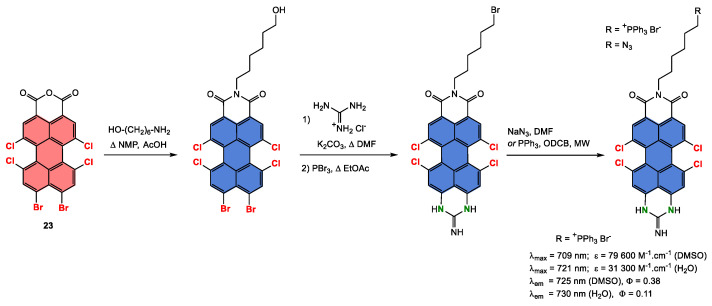
Synthesis of PMI dyes substituted with a guanidyl group in peri positions.

The 1,6,7,12-tetrachloro-9,10-dibromoPMI building block **23** was exploited to connect via a periannulation reaction a guanidyl group to furnish a D-π-A system (Scheme 13) [153]. In particular, a triphenylphosphonium group was grafted in the imide terminal position in order to combine the selectivity of triphenylphosphine toward mitochondria and the excellent photochemical properties of such dyes. These chromophores possess excellent water solubility and a maximum absorption around 710 nm, and they emit in the NIR region at 725 nm. After rapid cellular uptake, this PMI dye possessing a phosphonium group selectively stains mitochondria, which remains the universal target in cancer cells.

**Figure 35:**
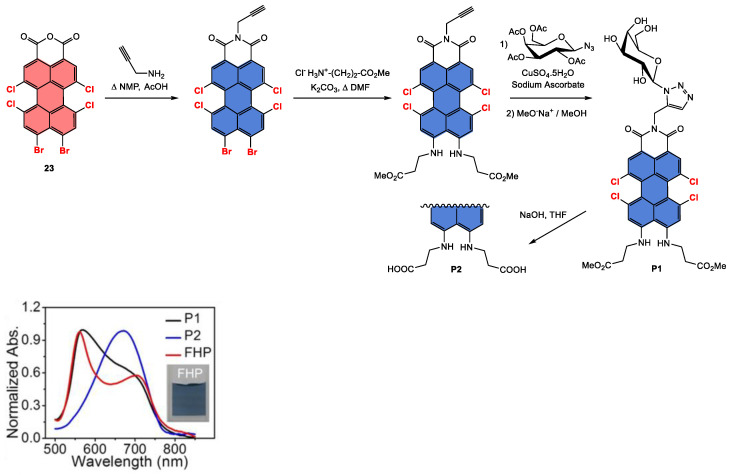

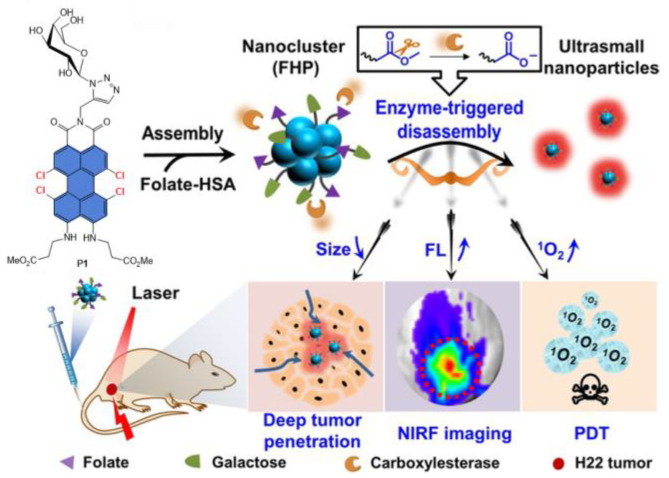
Synthesis of PMI derivatives decorated with a galactose unit and their normalized absorption spectra; illustrations of FHP-nanocluster fabrication, enzyme-triggered disassembly for deep-tumour penetration, near-infrared fluorescence imaging and photodynamic therapy. Adapted and reprinted with permission from reference [154]. Copyright 2020 Wiley-VCH GmbH.

This strategy was exploited to synthesize a carboxylesterase-responsive precursor by modifying the PMI core using the imidization with propargylamine, then double nucleophilic substitution in the peri positions, with β-alanine methylester groups playing the role of electron-donating groups. The hydrophilic galactose was grafted using a Huisgen azide-alkyne click reaction, producing the NIR-absorbing amphiphile system **P1** (Figure 35) [154]. The latter was assembled with folate-decorated albumins into a nanocluster named FHP with a diameter of around 100 nm. This material, **P1,** was selectively hydrolysed by the carboxylesterase in the tumour site leading to **P2**, with the size of FHP decreasing from 100 nm to 10 nm to facilitate its deep-tumour penetration. A blue-shifted absorption band was observed for **P1** in water compared to that of **P2**, for which the maximum absorption is around 700 nm, as the result of the aggregation of compound **P1** in water. Concomitantly, the enzyme-triggered disassembly of FHP enhanced NIR fluorescence and promoted singlet-oxygen generation to improve imaging-guided PDT.

**Scheme 14:**
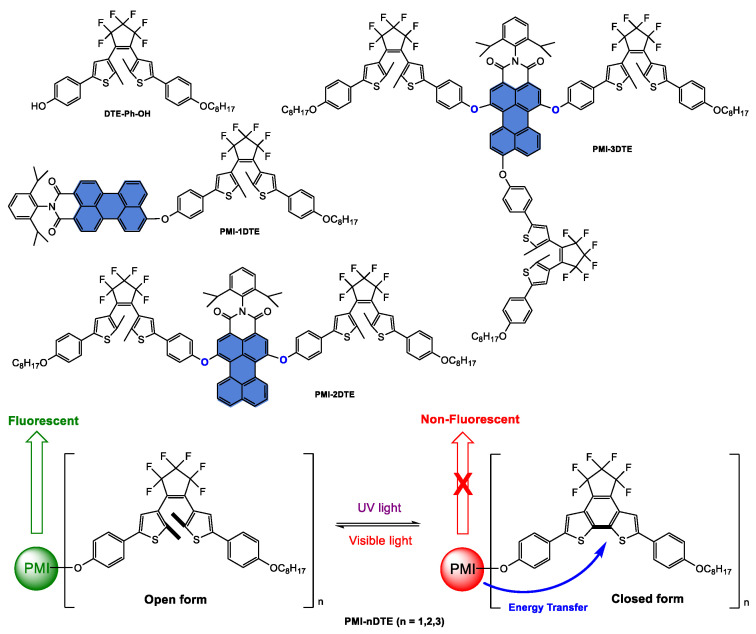
Synthesis of PMI-nDTE assemblies and mechanism of the photochromism and fluorescence switching occurring upon irradiation with visible and ultraviolet light.

Systems composed of one, two or three dithienylethene units (DTE) grafted on the PMI core were designed as a photoswitchable fluorophore for super-resolution imaging [155]. These assemblies were synthesized using a nucleophilic substitution with DTE-Ph-OH in the presence of K_2_CO_3_ on 9-bromoPMI **17** and 1,6,9-tribromoPMI **18**, producing **PMI-1DTE** and **PMI-3TDE**, respectively (Scheme 14). The triad **PMI-2TDE** was obtained by a selective nucleophilic substitution in both bay positions, starting from 1,6,9-tribromoPMI **18**. The debromination in the 9-peri position was performed using Pd(PPh_3_)_4_ as the catalyst in the presence of sodium carbonate in a mixture of DME:H_2_O. It was shown that the fluorescence inside these systems is reversibly quenched based on photochromic FRET from PMI to DTE units when the DTE units convert between the open- and the closed-ring isomers upon irradiation with visible and UV light, respectively.

**Scheme 15:**
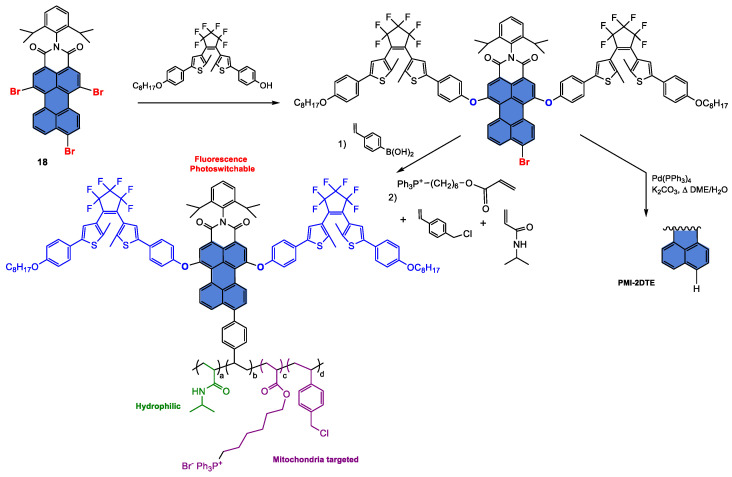
Synthesis of poly(PMI-2DTE)-co-VBC-co-TPP-co-NIPAM].

This strategy was used to synthesize water-soluble photoswitchable polyfluorophores based on the PMI–DTE system. The latter were designed to possess triphenylphosphonium and benzyl chloride, acting as two-fold mitochondria-targeting units [156]. On the other hand, the PMI unit played the role of a strong emitter and the two DTE units the role of photoswitching quencher. The incorporation of thermoresponsive N-isopropylacrylamide (NIPAM) afforded the hydrophilicity of these MitoTrackers poly(PMI-2DTE)-co-VBC-co-TPP-co-NIPAM] (Scheme 15).

**Figure 36:**
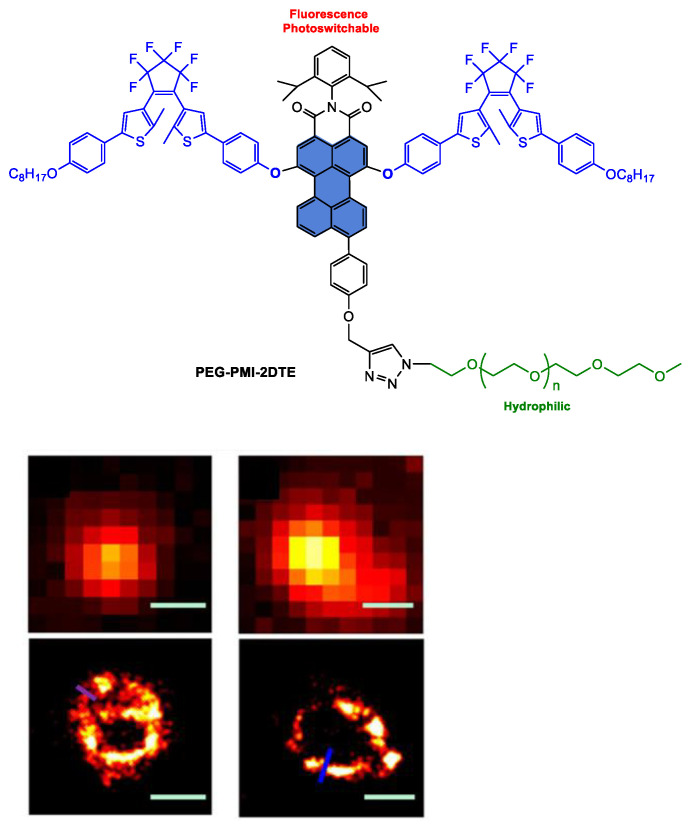
Structure of PEG-PMI-2DTE; fluorescence imaging (**top**) and super-resolution imaging (**bottom**) for liposomal vesicles and mitochondria. Scale-bar for all figures: 300 nm. Reprinted with permission from reference [157]. Copyright 2017 American Chemical Society.

These polyfluorophores with integrated functions exhibited the reversible fluorescence switching “on-state” and “off-state” in aqueous solutions by alternating the irradiation of UV light at 302 nm, which caused the cyclization of the DTE unit and visible light to reach the open state (Figure 36) [157]. They were used as mitochondria-targeted probes of HeLa cells to produce super-resolution imaging. The strategy proved to be versatile and was applied to synthesize an amphiphilic photoswitchable fluorescent probe, **PEG-PMI-2DTE**, by grafting PEG onto the PMI-2DTE unit using a Huisgen azide-alkyne click reaction. These fluorophores were used for the super-resolution fluorescence imaging of liposomal vesicles (multilamellar, large unilamellar and small unilamellar vesicles). Moreover, **PEG-PMI-2DTE** exhibited excellent fatigue-resistant photochromic properties along with fluorescence switching upon UV-light and visible-light irradiation, and the fine nanostructures of liposomes were observed under a super-resolution microscope with an optical resolution of 30 nm.

## 6. Conclusions

In this review, which aims to present recent applications of perylenediimide (PDI) and perylenemonoimide (PMI) derivatives for bioimaging and photothermal or photodynamic therapy applications, in the scope of these fantastic developments, we wanted to highlight the essential role and the fundamental work of organic chemists to design and synthesize systems that are always more original than the last. It is, indeed, important to show that molecular engineering is able to modulate the optical properties of these high-potential structures. In summary, we presented a broad update on the recent use of PDI- and PMI-based fluorophores for bioimaging, but also in photothermal therapy and photodynamic therapy, which are particularly important as suitable therapeutic alternatives with several advantages over traditional clinical approaches to cancer treatment. To achieve these goals, PDI- and PMI-based fluorophores must meet specifications in which solubility in water or biologically compatible solvents and absorption in the near-infrared region are particularly sought after. Thus, PDI- and PMI-based fluoroprobes, thanks to their broad absorption and strong emissive properties, can meet this challenge. The ability to modulate their electronic properties relatively easily by accurate modification in the ortho and bay positions, as well as the peri position for PMI, is a unique asset that remains to be further exploited. Moreover, these polyaromatic systems present quite good biocompatibility and low cytotoxicity, which are mandatory for practical biomedicinal applications. Of course, these PDI and PMI units are subject to aggregation by π–π interactions, but the introduction of hydrophilic groups can effectively prevent this aggregation, thus facilitating the generation and release of singlet oxygen, a condition for developing active systems for photodynamic therapy. On the other hand, the development of fluorescence probes with high selectivity for cellular imaging with suitable functionality has become an important research avenue. In this review, we have summarized the strategies recently used for designing PDI and PMI chromophores and their structure–property relationships for these targeted applications. One of the most critical issues in cancer theranostics is the limited light-penetration depth. To further increase transparency, these dyes with increased red-shifted absorption bands are required to attain the NIR-II region. Organic chemists will use their ingenuity to design new PDI- and PMI-based systems. Several routes can be considered, such as incorporating other strong electron-donating substituents, more intensive development of terrylene- and quaterrylene-based chromophores and more extended structures by using PMI and PDI backbones as starting materials, to address all these challenges.

## Data Availability

Not applicable.

## Abbreviations

All-polymer solar cells (All-PSCs); atom transfer radical polymerization (ATRP); benzoperylene monoimide (BPMI); camptothecin (CPT); cerium ammonium nitrate (CAN); β-cyclodextrin (CD); 1,8-diazabicyclo[5.4.0]undec-7-ene (DBU); 4-dimethylaminopyridine (DMAP); doxorubicin (DOX); drug-delivery system (DDS); enhanced permeability retention (EPR); ethyl ethylene phosphate (EEP); fluorescence resonance energy transfer (FRET); fluorescent supramolecular micelles (FSMs); folic acid (FA); glutathione (GSH); hexamethylene diisocyanate (HDI); highest occupied molecular orbital (HOMO); intersystem-crossing process (ISC); lower critical solution temperature (LCST); lowest unoccupied molecular orbital (LUMO); nanoparticles (NPs); near-infrared (NIR); near-infrared fluorescence (NIRF); nonfullerene acceptors (NFAs); oligoethylene glycol (OEG); organic field-effect transistors (OFETs); organic light-emitting diodes (OLEDs); organic photovoltaics (OPVs); organic solar cells (OSCs); pentafluorophenyl (PFP); perfluorocarbon (PFC); perylenebisimide (PBI); 3;4-perylenedicarboxylic acid monoanhydride (PDCMA); perylenediimide (PDI); perylenediimide acrylate (PDA); perylenemonoimide (PMI); photoluminescence (PL); photothermal conversion efficiency (PCE); perylene-3;4:9;10-tetracarboxylic acid dianhydride (PTCDA); phosphate-buffered saline (PBS); photoacoustic (PA); photoacoustic imaging (PAI); photodynamic therapy (PDT); photoinduced electron transfer (PET); photosensitizer (PS); photothermal therapy (PTT); polycaprolactone (PCL); polycyclic aromatic hydrocarbons (PAHs); poly(ethylene glycol) (PEG); poly(ethyleneglycol) methacrylate (PEGMA); photoinduced electron transfer (PET); poly(ethylenimine)-g-poly(lactide-co-glycolide)-g-poly(ethylenimine) (PLGA-PEI); polymer nanoparticles (PNPs); poly(N-isopropylacrylamide (PNIPAM); quaterrylenediimide (QDI); reactive oxygen species (ROS); reversible addition fragmentation transfer (RAFT); ring-opening polymerization (ROP); shell-crosslinked nanoparticles (SCNPs); silicon quantum dots (SQDs); supramolecular drug-delivery system (SDDS); terrylenediimide (TDI); thermosensitive polymer (TP); ultrasound imaging (USI).
